# Supplementary material for: Activation of LXR Receptors and Inhibition of TRAP1 Causes Synthetic Lethality in Solid Tumors
Source: Cancers (Basel). 2019 Jun 7;11(6):788. doi: 10.3390/cancers11060788 (PMC6627953; doi:10.3390/cancers11060788)
Supplement: Supplementary file 1 [file cancers-11-00788-s001.zip › cancers-512184-supplementary materials/cancers-512184-suppl-final-checked.pdf]

Supplementary Materials: Activation of LXR Receptors and Inhibition of TRAP1 Causes Synthetic Lethality in Solid Tumors

Trang Thi Thu Nguyen, Chiaki Tsuge Ishida, Enyuan Shang, Chang Shu, Elena Bianchetti, Georg Karpel-Massler and Markus D. Siegelin

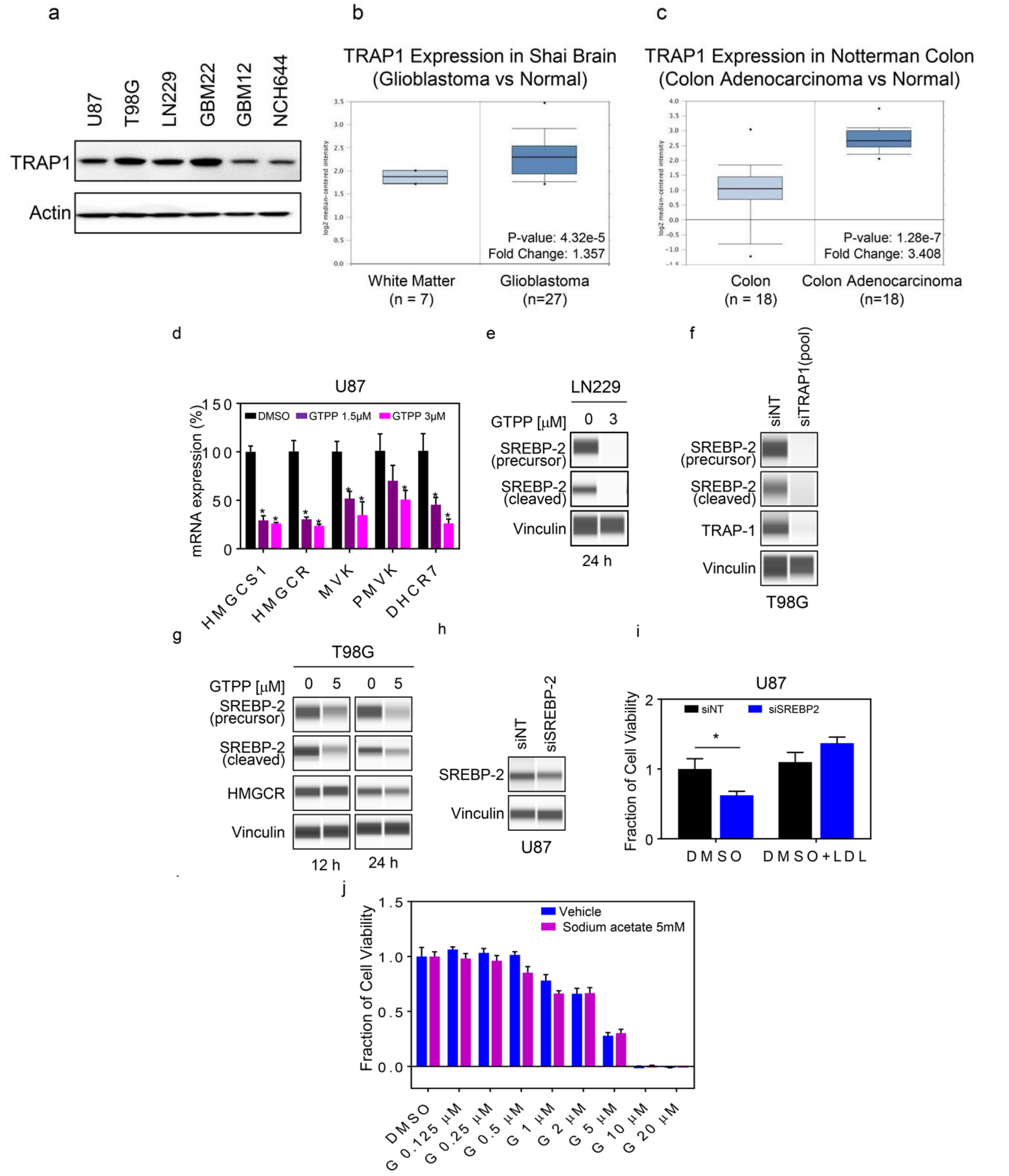

Figure S1. Gamitrinib suppresses the levels of SREBP2 and HMGCR. (**a**) The TRAP1 protein expression in GBM cell lines; (**b**) Oncomine database to demonstrate that TRAP1 levels are elevated in glioma samples as

compared to normal brain tissue; (**c**) Oncomine database to demonstrate that TRAP1 levels are elevated in Colon Adenocarcinoma samples as compared to normal Colon; (**d**) Real time PCR analysis of U87 cells were treated with 1.5 µM or 3 µM GTPP for 24h. Shown are means and SD ($n \geq 4$); **(e)** LN229 cells were treated with 3 µM GTPP for 24h. Thereafter, lysates were collected and analyzed for the expression of SREBP2 (precursor) and SREBP2 (cleaved). Vinculin was used as loading control; **(f)**, T98G cells were transfected with siNT or siTRAP1 (pool) for 72h. Thereafter, lysates were collected and analyzed for the expression of SREBP2 (precursor), SREBP2 (cleaved) and TRAP1; **(g)** T98G cells were treated with 5 µM GTPP for 12h and 24h. Thereafter, lysates were collected and analyzed for the expression of SREBP2 (precursor), SREBP2 (cleaved) and HMGCR; **(h,i)** U87 GBM cells were transfected with siRNA against SREBP2 for 72h. Thereafter, cells were exposed to a cellular viability assays for another 72h in the presence or absence of LDL A cellular viability assays was performed. Shown are means and SD ($n \geq 4$); **(j)** U87 GBM cells were treated with increasing concentrations of gamitrinib in the presence or absence sodium acetate for 72h. A cellular viability assays was performed. Shown are means and SD ($n \geq 4$). $* p < 0.05$.

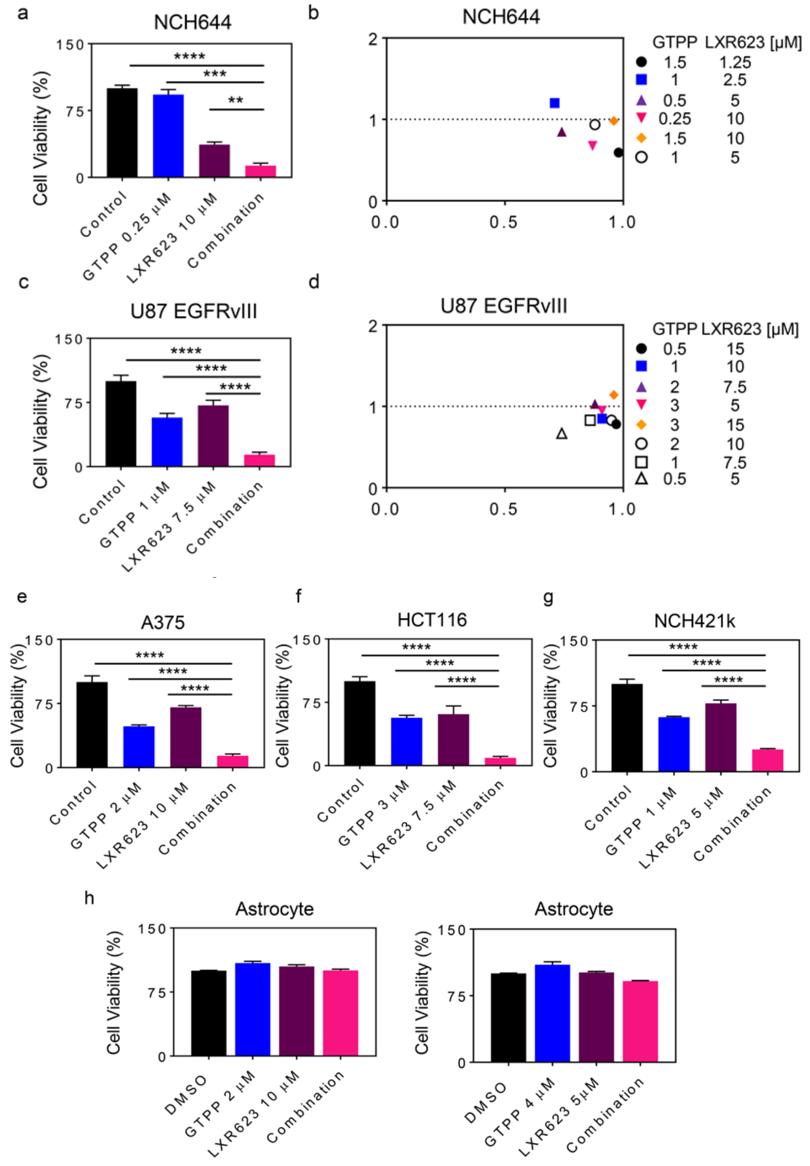

Figure S2. Synergistic reduction in cellular viability in solid tumor cancer cells. **(a,c)** NCH644 and U87-EGFRvIII cells were treated with the indicated concentrations of GTPP, LXR623 or the combination of both for 72h. Thereafter, cellular viability was analyzed and statistical analysis was performed. Shown are means and SD ($n \geq 4$)**; (b,d)** CI (combination index) value indicates as to whether the drug combination is either additive (CI value = 1.0), synergistic (CI value < 1.0) or antagonistic (CI value > 1.0). The dotted line represents additivity with respect to the combination treatment; **(e–h)** A375, HCT116, NCH421k and Astrocytes were treated with the indicated concentrations of GTPP, LXR623 or the combination of both for 72h. Thereafter, cellular viability was analyzed and statistical analysis was performed. Shown are means and SD ($n \geq 4$). **** $p < 0.001$.

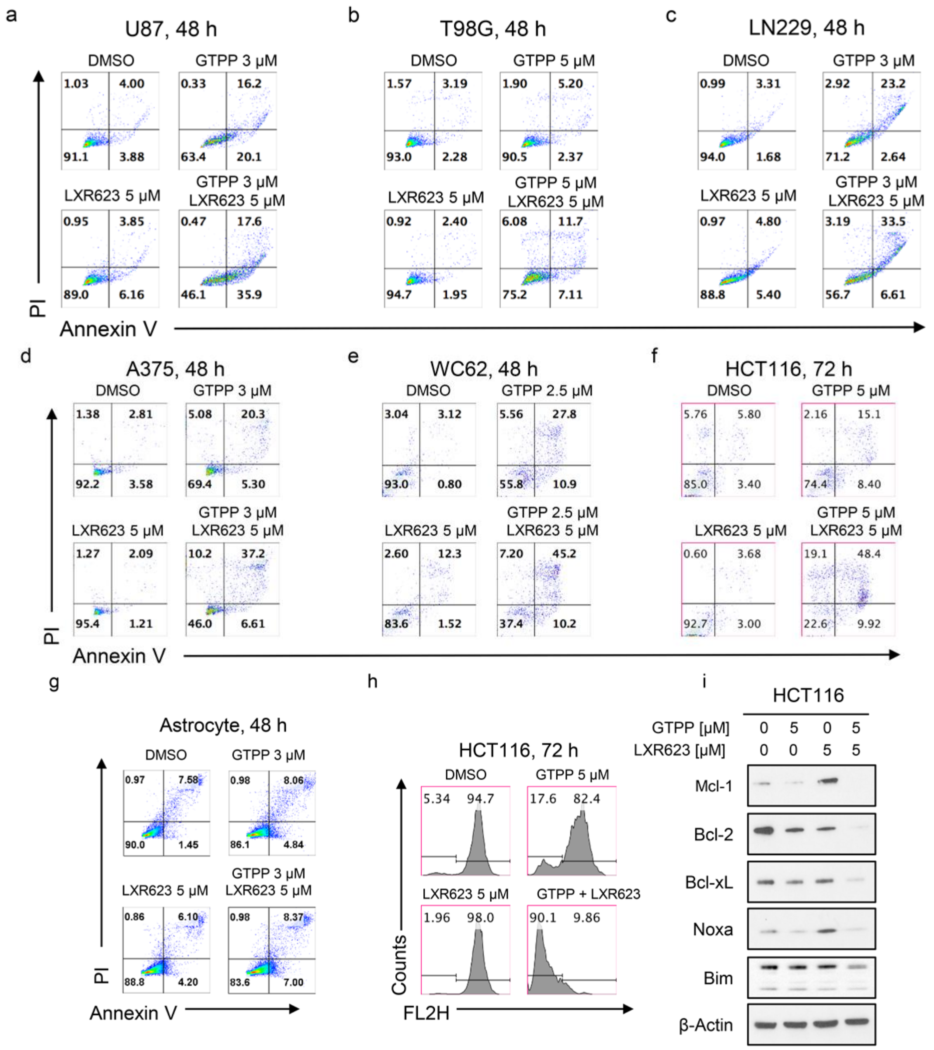

Figure S3. The combination treatment of gamitrinib and LXR623 causes synergistic cell death with apoptotic features. **(a–g)** U87, T98G, LN229, A375, WC62, HCT116 and Astrocyte cells were treated with the indicated concentrations of GTPP, LXR623 or the combination of both. Thereafter, cells were stained with annexin V/propidium iodide and analyzed by multi-parametric flow cytometry; (**h**) HCT116 (72h treatment) were treated with 3 μM GTPP, 5 μM LXR623 or the combination of both, stained with TMRE and analyzed by flow cytometric analysis for dissipation of mitochondrial membrane potential; (**i**) HCT116 cells were treated with the indicated concentrations of GTPP, LXR623, or combination of both for 72 h. Thereafter, protein lysates were prepared and analyzed for the expression of Mcl-1, Bcl-2, Bcl-xL, Noxa, BIM and Vinculin.

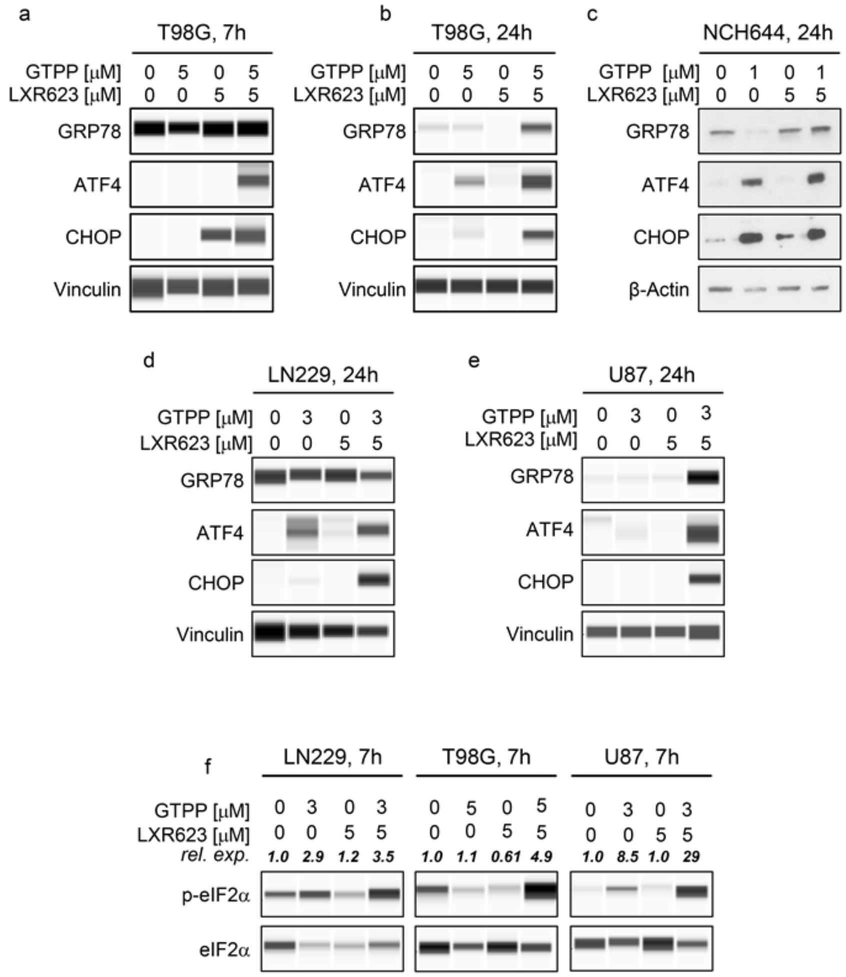

Figure S4. Enhanced activation of endoplasmic reticulum stress elicited by the combination treatment of gamitrinib and LXR623. **(a,b)** T98G cells were treated with 5 μM GTPP, 5 μM LXR623 or combination of both for 7h and 24h. Thereafter, protein lysates were prepared and analyzed for the expression of GRP78, ATF4, CHOP, and Vinculin. Vinculin was used as loading control; **(c)** NCH644 cells were treated with 1 μM GTPP, 5 μM LXR623 or combination of both for 24h. Thereafter, protein lysates were prepared and analyzed for the expression of GRP78, ATF4, CHOP, and Vinculin; **(d,e)** LN229 and U87 cells were treated with 3 μM GTPP, 5 μM LXR623 or combination of both for 24h. Thereafter, protein lysates were prepared and analyzed for the expression of GRP78, ATF4, CHOP, and Vinculin; (**f**) LN229, T98G and U87 were treated with indicated concentration of GTPP and LXR623 for 7h. Protein lysates were prepared and analyzed for the expression of p-eIF2*α* and eIF2*α*. The expression levels of p-eIF2*α* (serine 51) over eIF2*α* were quantified by using ImageJ (shown in cursive font).

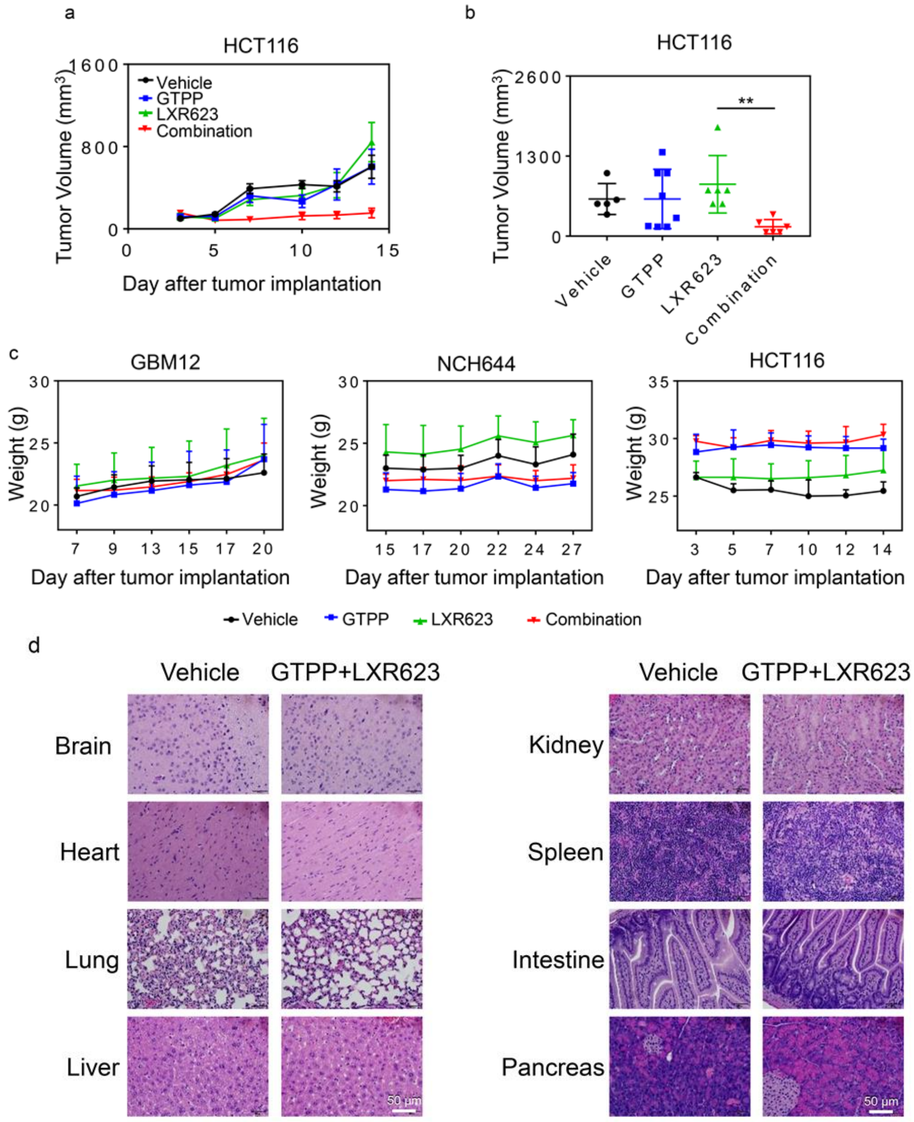

Figure S5. The combination treatment of gamitrinib and LXR623 reduces tumor growth more efficiently than single treatments in solid xenograft tumor models. **(a)** 1 × 10^6 HCT116 cells were implanted subcutaneously. After tumor formation, groups were formed. Animals were treated intraperitoneally with vehicle, LXR623 (200 mg/kg), GTPP (5 mg/kg) or both agents (six treatments). Tumor growth curves show the development of tumor size for each treatment group. Shown are means and SD ($n \geq 5$); **(b)** Scatter plots display the quantitative representation of the tumor size among the different treatments at the end of the experiment. Shown are means and SD ($n \geq 5$); (**c**) Body weight of mice that were implanted with GBM12 PDX tissue, NCH644 cells and HCT116 cells and treated as indicated. Shown are means and SD ($n \geq 5$); **(d)** Representative histopathological images (hematoxylin and eosin stain) are shown in the vehicle and the GTPP and LXR623 combination treatment of different organelle related to the GBM12 xenograft model in Figure 6. ** $p < 0.01$.
