# Supplementary material for: Activation of LXR Receptors and Inhibition of TRAP1 Causes Synthetic Lethality in Solid Tumors
Source: Cancers (Basel). 2019 Jun 7;11(6):788. doi: 10.3390/cancers11060788 (PMC6627953; doi:10.3390/cancers11060788)

Supplementary Materials: Activation of LXR Receptors and Inhibition of TRAP1 Causes Synthetic Lethality in Solid Tumors

Trang Nguyen, Chiaki Ishida, Enyuan Shang, Chang Shu, Elena Bianchetti, Georg Karpel-Massler and Markus Siegelin

Uncropped blot and capillary electrophoresis images

Figure 1e

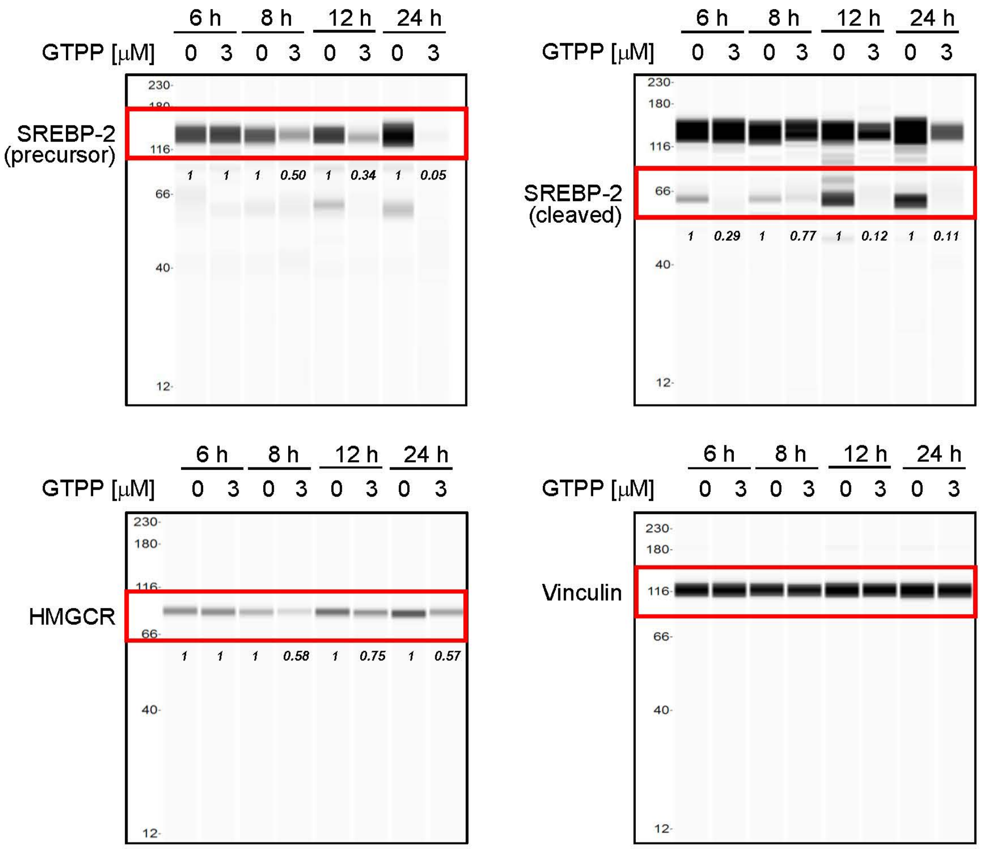
6 h 8 h 12 h 24 h
GTPP [μM] 0 3 0 3 0 3 0 3
SREBP-2 (precursor)
1 1 1 0.50 1 0.34 1 0.05
SREBP-2 (cleaved)
1 0.29 1 0.77 1 0.12 1 0.11
HMGCR
1 1 1 0.58 1 0.75 1 0.57
Vinculin
230
180
116
66
40
12

Figure 1f

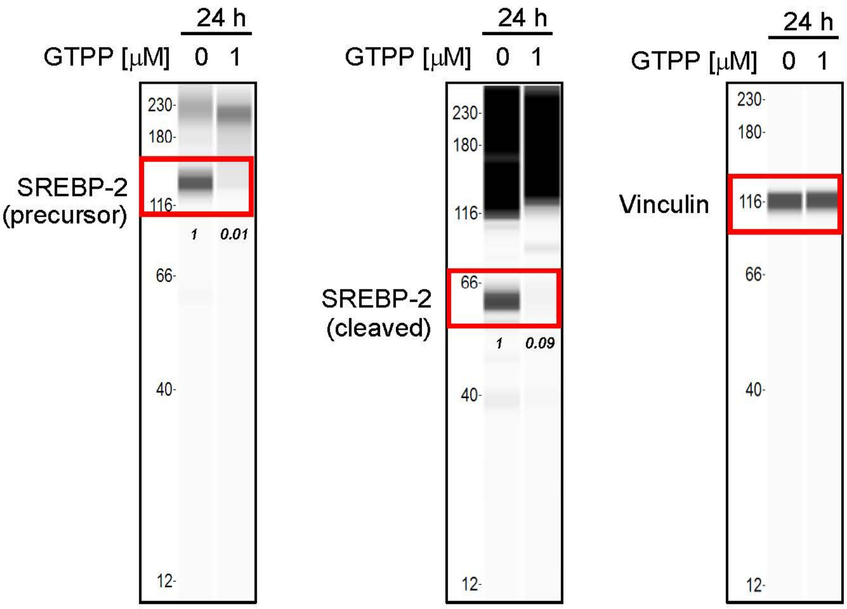
24 h
GTPP [μM] 0 1
230-
180-
SREBP-2
(precursor)
116-
1 0.01
66-
40-
12-
24 h
GTPP [μM] 0 1
230-
180-
116-
66-
SREBP-2
(cleaved)
1 0.09
40-
12-
24 h
GTPP [μM] 0 1
230-
180-
Vinculin
116-
66-
40-
12-

Figure 1g

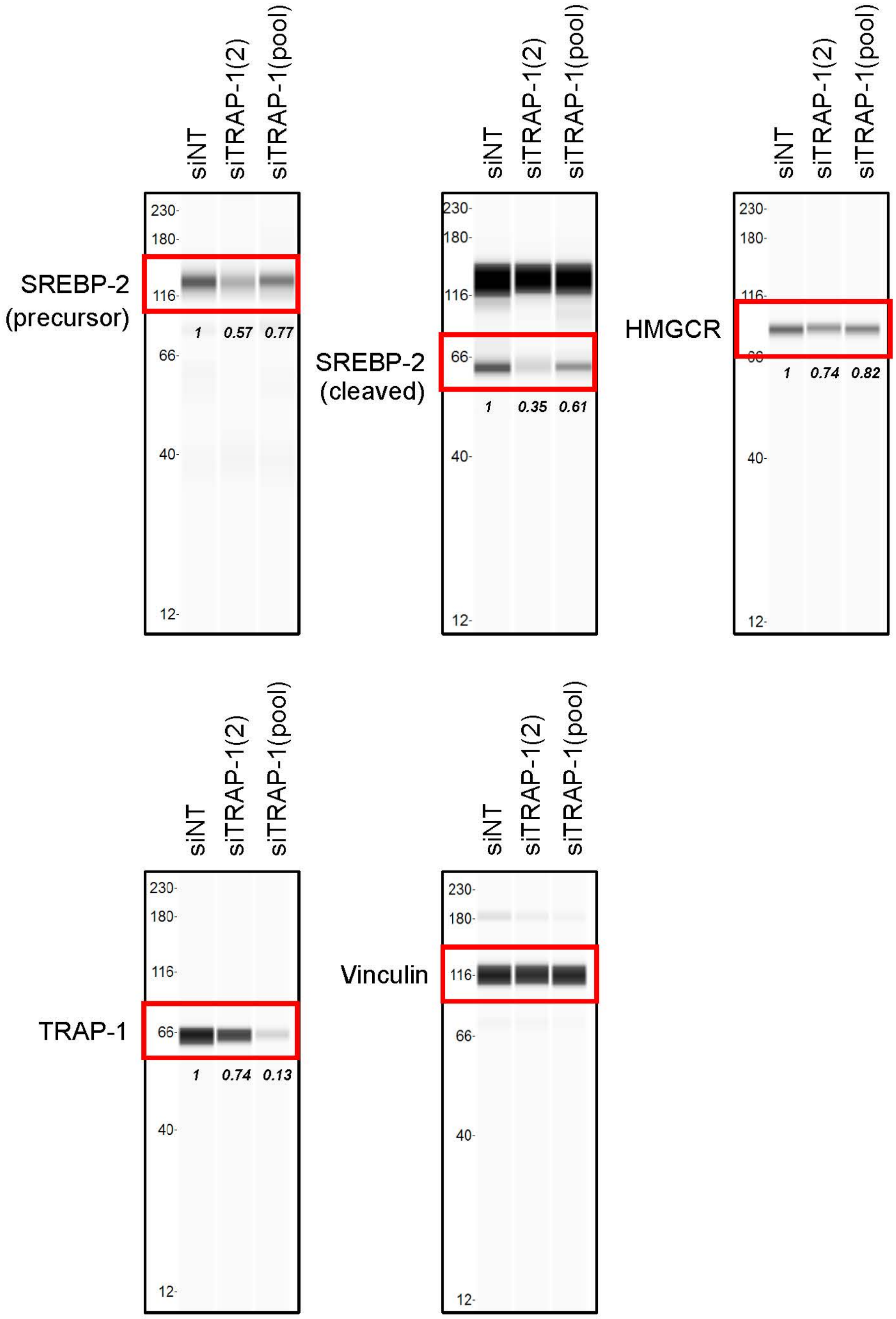
siNT
siTRAP-1(2)
siTRAP-1(pool)
SREBP-2
(precursor)
1 0.57 0.77
SREBP-2
(cleaved)
1 0.35 0.61
HMGCR
1 0.74 0.82
TRAP-1
1 0.74 0.13
Vinculin
230
180
116
66
40
12

Figure 1h

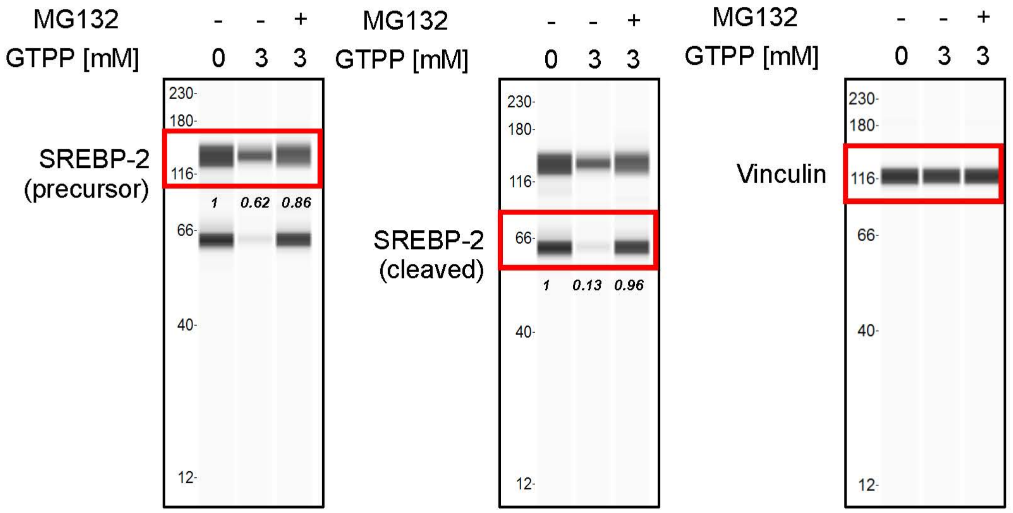
MG132
GTPP [mM]
- - +
0 3 3
230
180
116
66
40
12
SREBP-2
(precursor)
1 0.62 0.86
SREBP-2
(cleaved)
1 0.13 0.96
Vinculin

Figure 1i

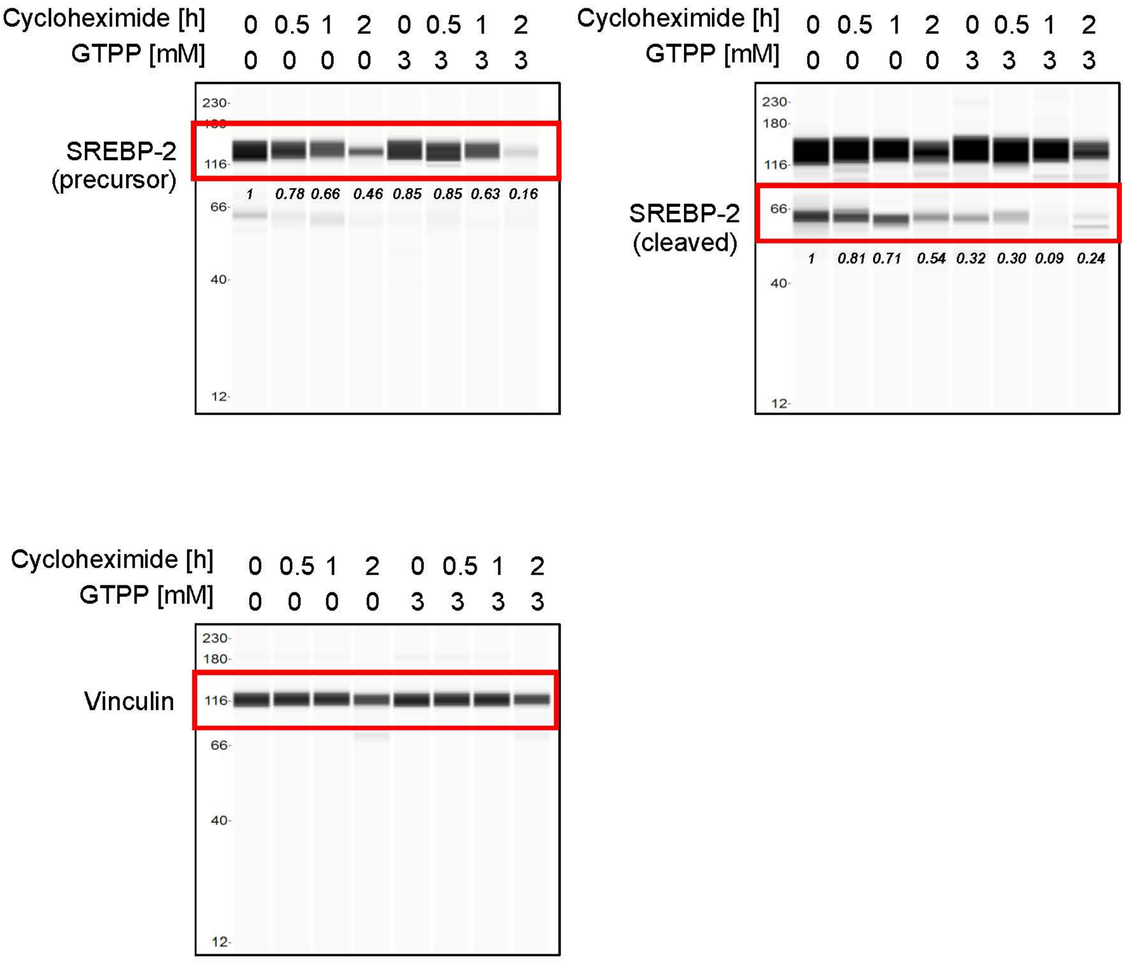
Cycloheximide [h] 0 0.5 1 2 0 0.5 1 2
GTPP [mM] 0 0 0 0 3 3 3 3
230
180
116
66
40
12
SREBP-2 (precursor)
1 0.78 0.66 0.46 0.85 0.85 0.63 0.16
Cycloheximide [h] 0 0.5 1 2 0 0.5 1 2
GTPP [mM] 0 0 0 0 3 3 3 3
230
180
116
66
40
12
SREBP-2 (cleaved)
1 0.81 0.71 0.54 0.32 0.30 0.09 0.24
Cycloheximide [h] 0 0.5 1 2 0 0.5 1 2
GTPP [mM] 0 0 0 0 3 3 3 3
230
180
116
66
40
12
Vinculin

Figure 1j

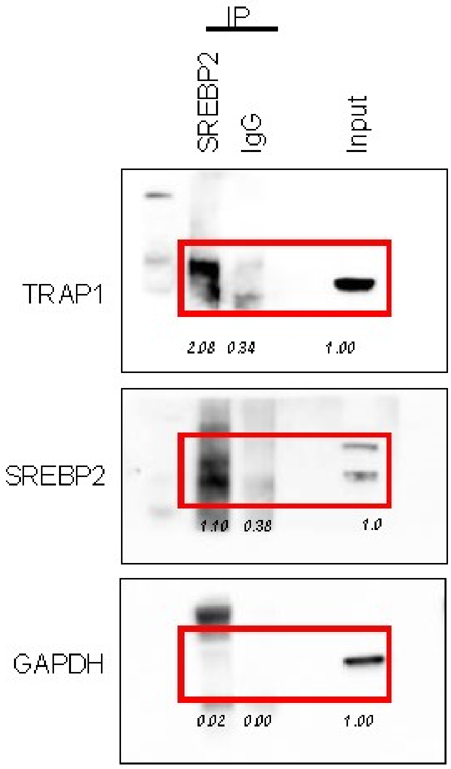
IP
SREBP2
IgG
Input
TRAP1
2.08 0.34 1.00
SREBP2
1.10 0.38 1.0
GAPDH
0.02 0.00 1.00

Figure 3f

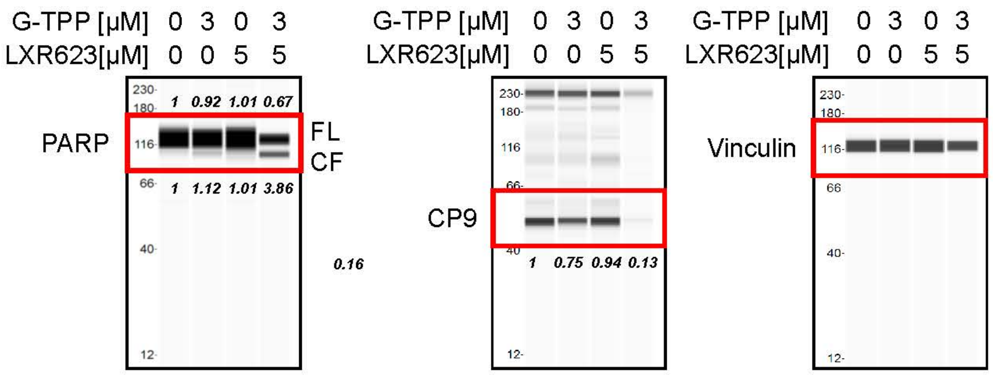
G-TPP [μM] 0 3 0 3
LXR623[μM] 0 0 5 5
PARP
1 0.92 1.01 0.67
FL
CF
1 1.12 1.01 3.86
0.16
G-TPP [μM] 0 3 0 3
LXR623[μM] 0 0 5 5
CP9
1 0.75 0.94 0.13
G-TPP [μM] 0 3 0 3
LXR623[μM] 0 0 5 5
Vinculin
230
180
116
66
40
12

Figure 4a

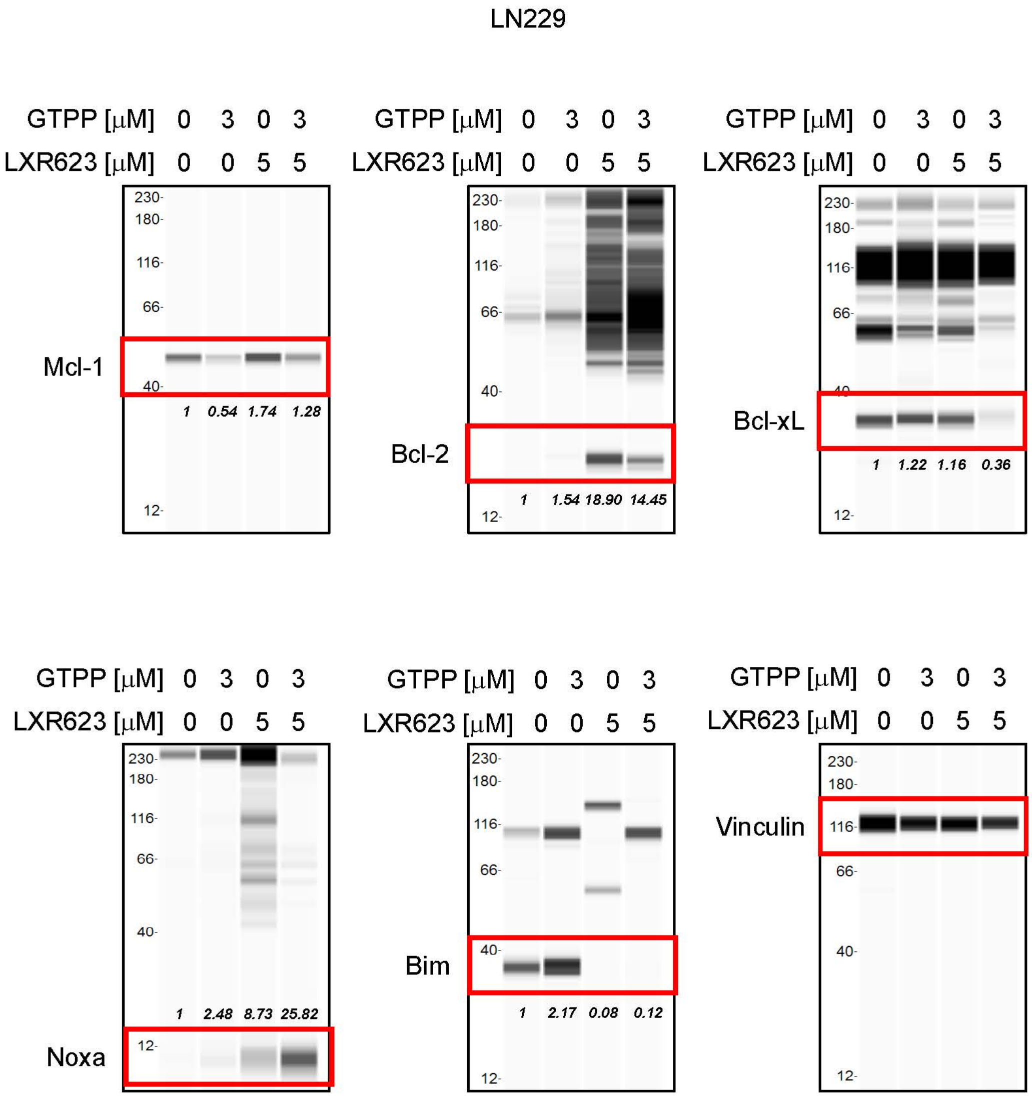
LN229
GTPP [μM] 0 3 0 3
LXR623 [μM] 0 0 5 5
230-
180-
116-
66-
40-
12-
Mcl-1
1 0.54 1.74 1.28
Bcl-2
1 1.54 18.90 14.45
Bcl-xL
1 1.22 1.16 0.36
Noxa
1 2.48 8.73 25.82
Bim
1 2.17 0.08 0.12
Vinculin

Figure 4a

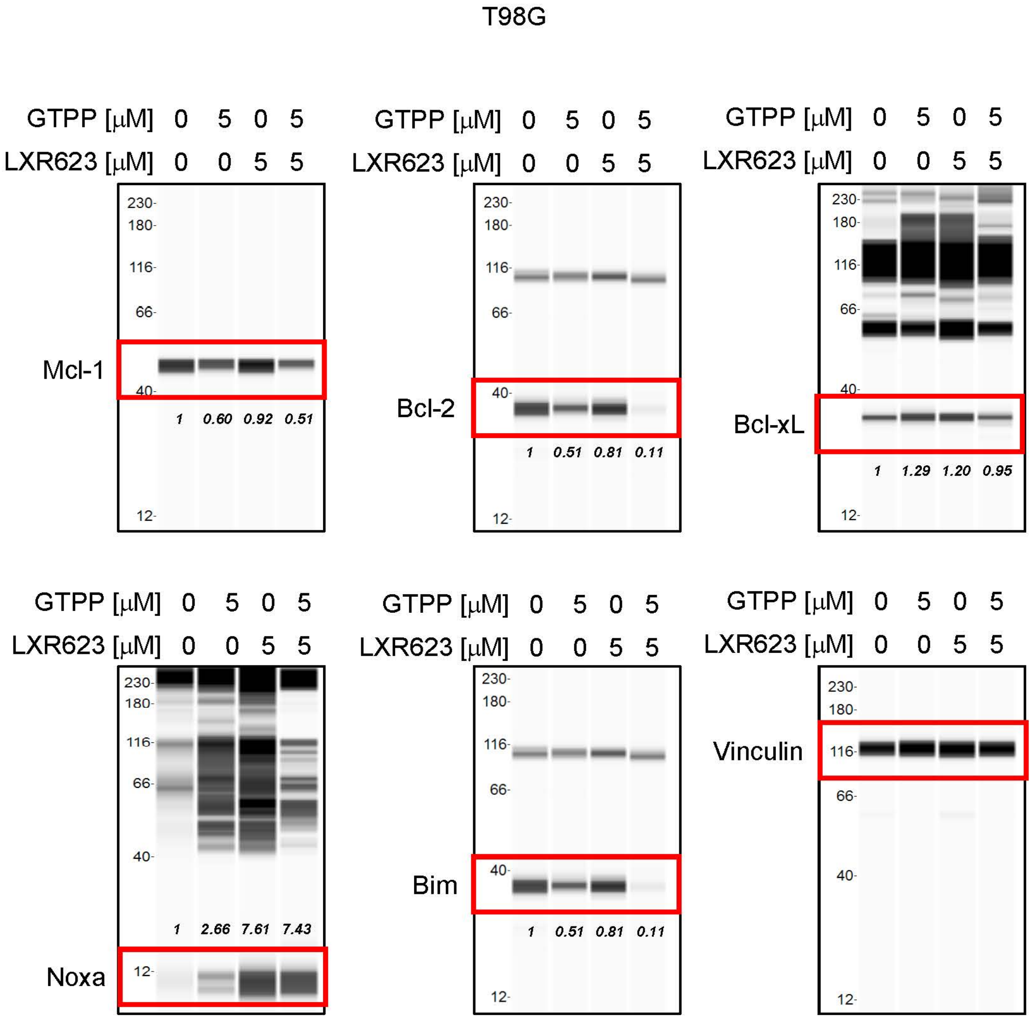
T98G
GTPP [μM] 0 5 0 5
LXR623 [μM] 0 0 5 5
Mcl-1
1 0.60 0.92 0.51
Bcl-2
1 0.51 0.81 0.11
Bcl-xL
1 1.29 1.20 0.95
Noxa
1 2.66 7.61 7.43
Bim
1 0.51 0.81 0.11
Vinculin
230
180
116
66
40
12

Figure 4a

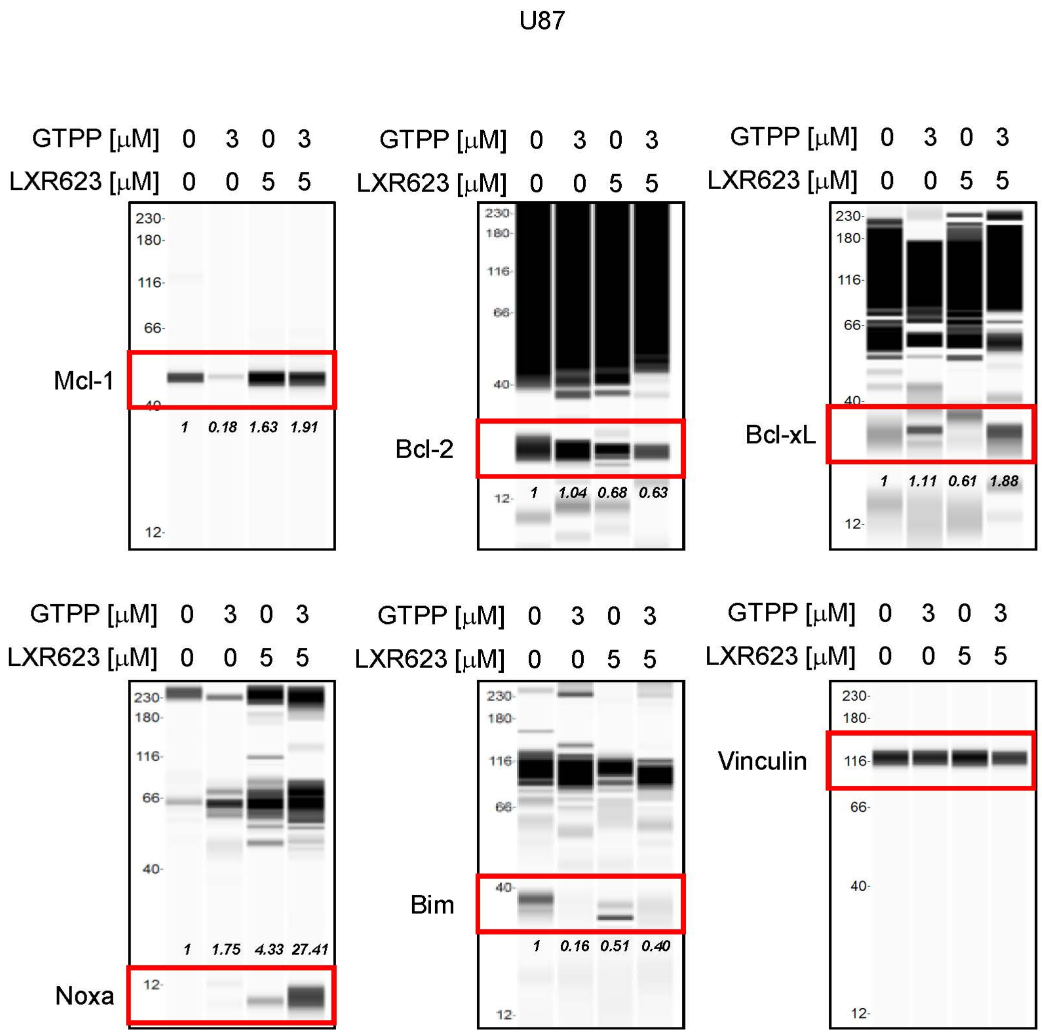
U87
GTPP [μM] 0 3 0 3
LXR623 [μM] 0 0 5 5
Mcl-1
1 0.18 1.63 1.91
Bcl-2
1 1.04 0.68 0.63
Bcl-xL
1 1.11 0.61 1.88
Noxa
1 1.75 4.33 27.41
Bim
1 0.16 0.51 0.40
Vinculin
230
180
116
66
40
12

Figure 4a

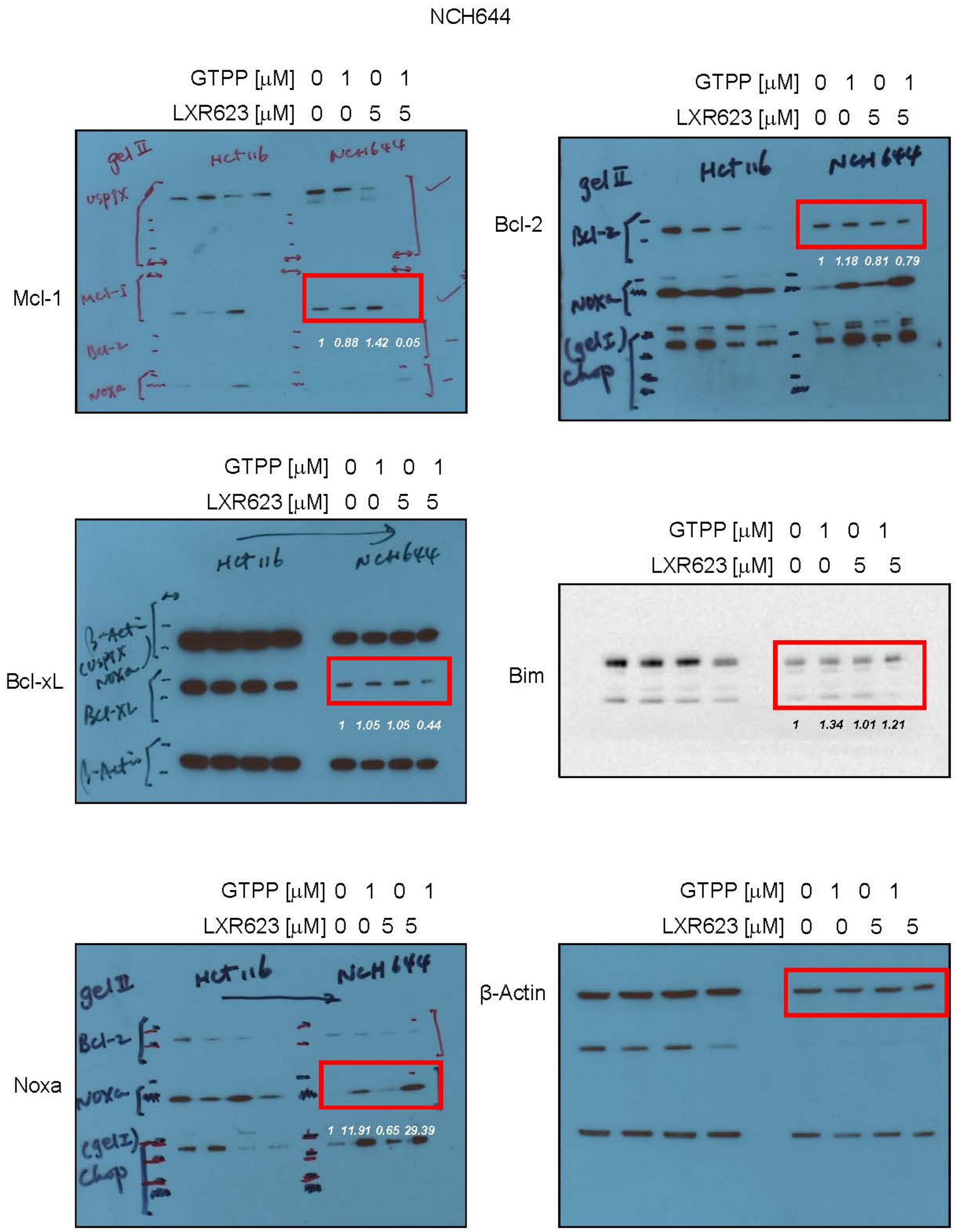
NCH644
GTPP [µM] 0 1 0 1
LXR623 [µM] 0 0 5 5
Mcl-1
1 0.88 1.42 0.05
GTPP [µM] 0 1 0 1
LXR623 [µM] 0 0 5 5
Bcl-2
1 1.18 0.81 0.79
GTPP [µM] 0 1 0 1
LXR623 [µM] 0 0 5 5
Bcl-xL
1 1.05 1.05 0.44
GTPP [µM] 0 1 0 1
LXR623 [µM] 0 0 5 5
Bim
1 1.34 1.01 1.21
GTPP [µM] 0 1 0 1
LXR623 [µM] 0 0 5 5
Noxa
1 11.91 0.65 29.39
GTPP [µM] 0 1 0 1
LXR623 [µM] 0 0 5 5
β-Actin

Figure 4c

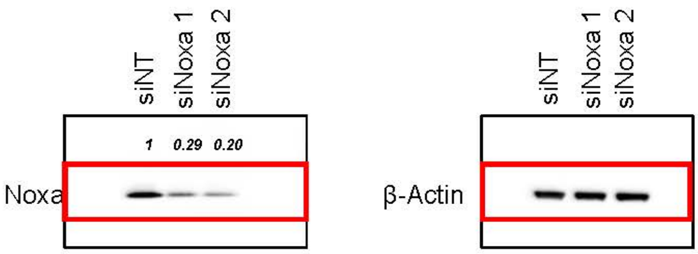
siNT
siNoxa 1
siNoxa 2
1
0.29
0.20
Noxa
siNT
siNoxa 1
siNoxa 2
β-Actin

Figure 4d

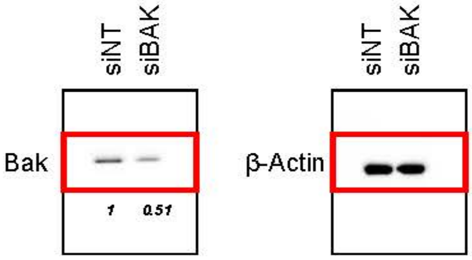

Figure 5d

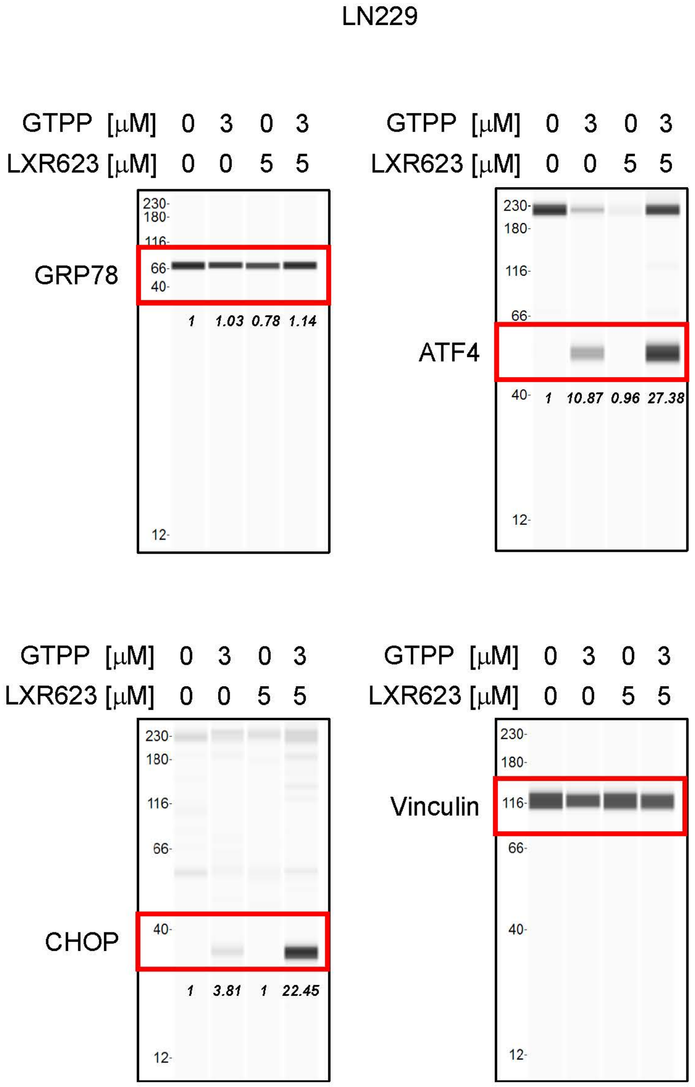
LN229
GTPP [µM] 0 3 0 3
LXR623 [µM] 0 0 5 5
GRP78
1 1.03 0.78 1.14
GTPP [µM] 0 3 0 3
LXR623 [µM] 0 0 5 5
ATF4
1 10.87 0.96 27.38
GTPP [µM] 0 3 0 3
LXR623 [µM] 0 0 5 5
CHOP
1 3.81 1 22.45
GTPP [µM] 0 3 0 3
LXR623 [µM] 0 0 5 5
Vinculin
230
180
116
66
40
12

Figure 5d

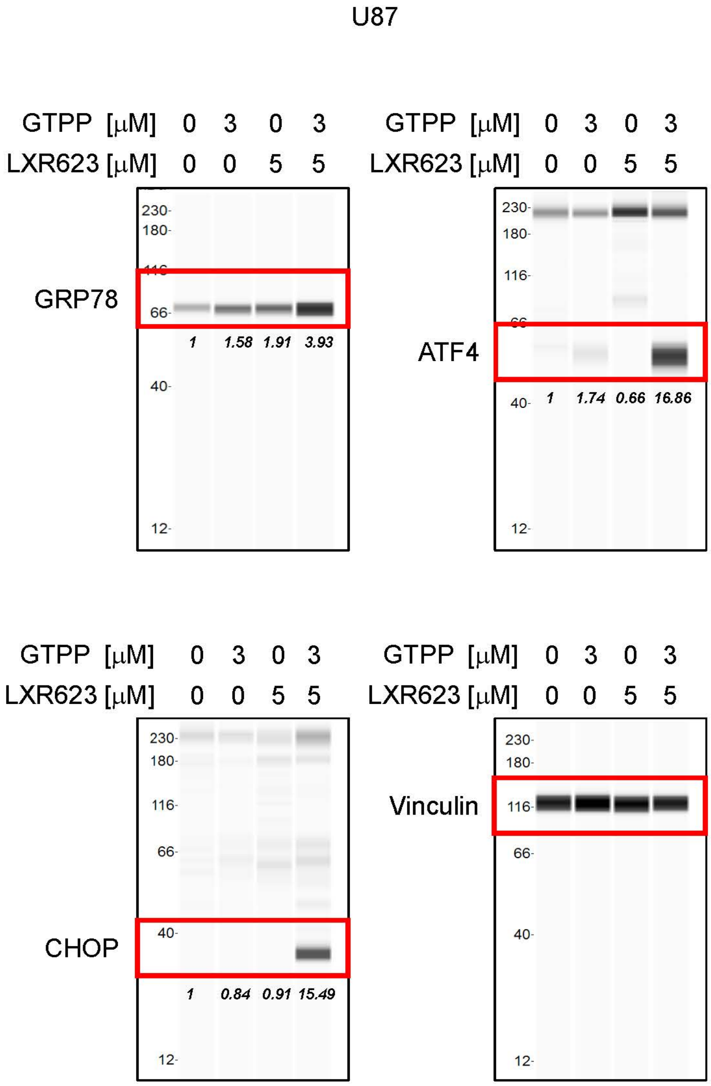
U87
GTPP [μM] 0 3 0 3
LXR623 [μM] 0 0 5 5
GRP78
230-
180-
66-
40-
12-
1 1.58 1.91 3.93
GTPP [μM] 0 3 0 3
LXR623 [μM] 0 0 5 5
ATF4
230-
180-
116-
40-
12-
1 1.74 0.66 16.86
GTPP [μM] 0 3 0 3
LXR623 [μM] 0 0 5 5
CHOP
230-
180-
116-
66-
40-
12-
1 0.84 0.91 15.49
GTPP [μM] 0 3 0 3
LXR623 [μM] 0 0 5 5
Vinculin
230-
180-
116-
66-
40-
12-

Figure 5e

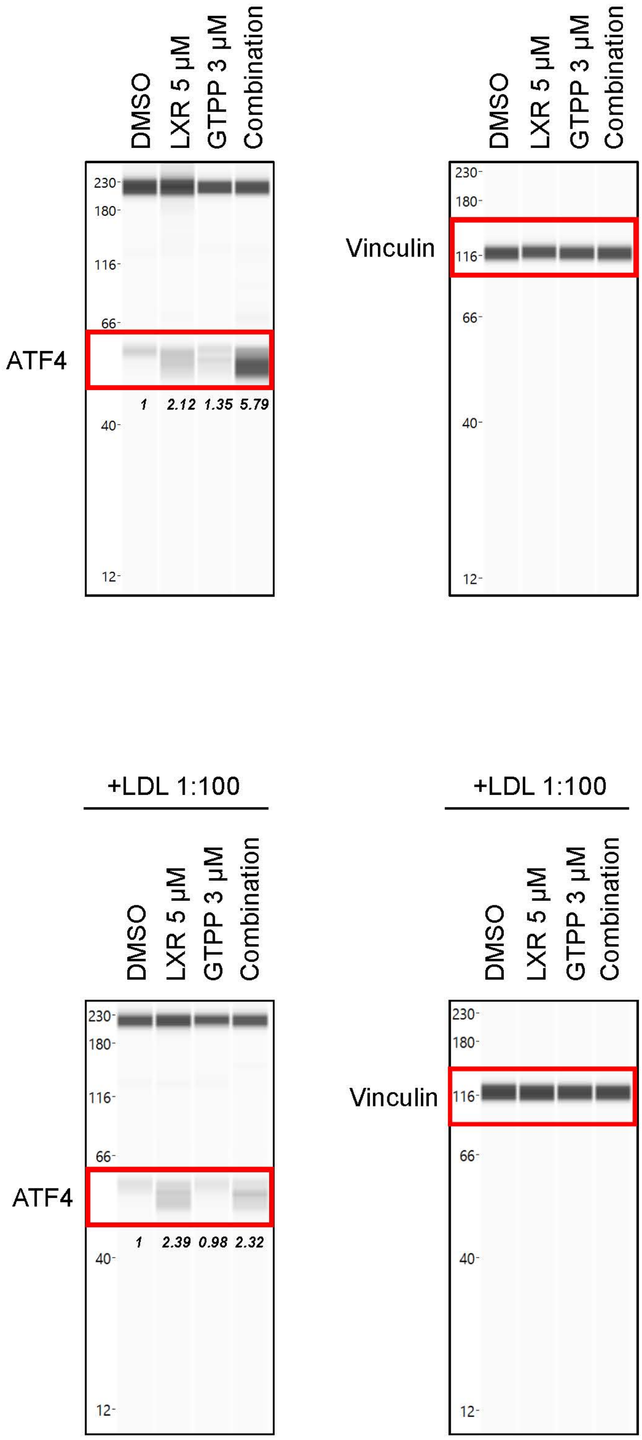
DMSO
LXR 5 µM
GTPP 3 µM
Combination
230
180
116
66
ATF4
1
2.12
1.35
5.79
40
12
Vinculin
+LDL 1:100
2.39
0.98
2.32

Figure 5f

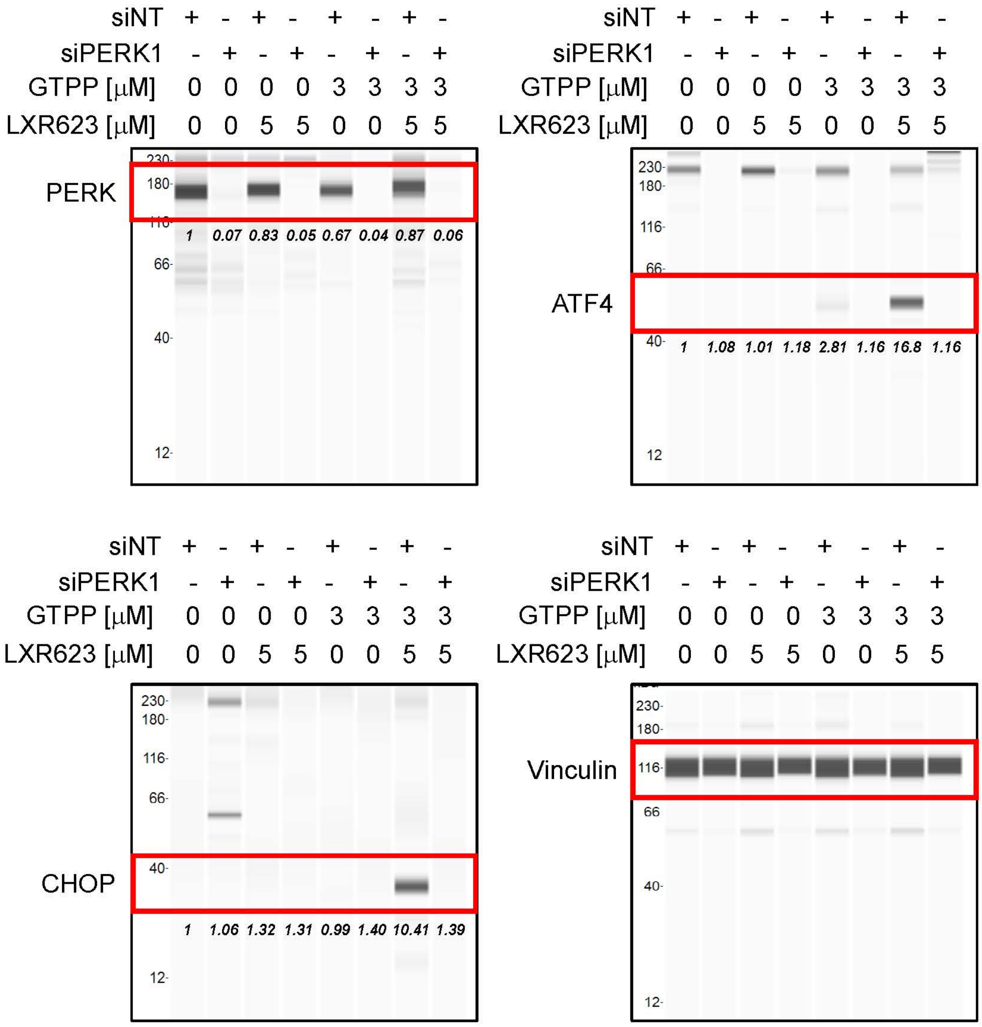
siNT + - + - + - + -
siPERK1 - + - + - + - +
GTPP [μM] 0 0 0 0 3 3 3 3
LXR623 [μM] 0 0 5 5 0 0 5 5
PERK
1 0.07 0.83 0.05 0.67 0.04 0.87 0.06
ATF4
1 1.08 1.01 1.18 2.81 1.16 16.8 1.16
CHOP
1 1.06 1.32 1.31 0.99 1.40 10.41 1.39
Vinculin
230
180
116
66
40
12

Figure 5g

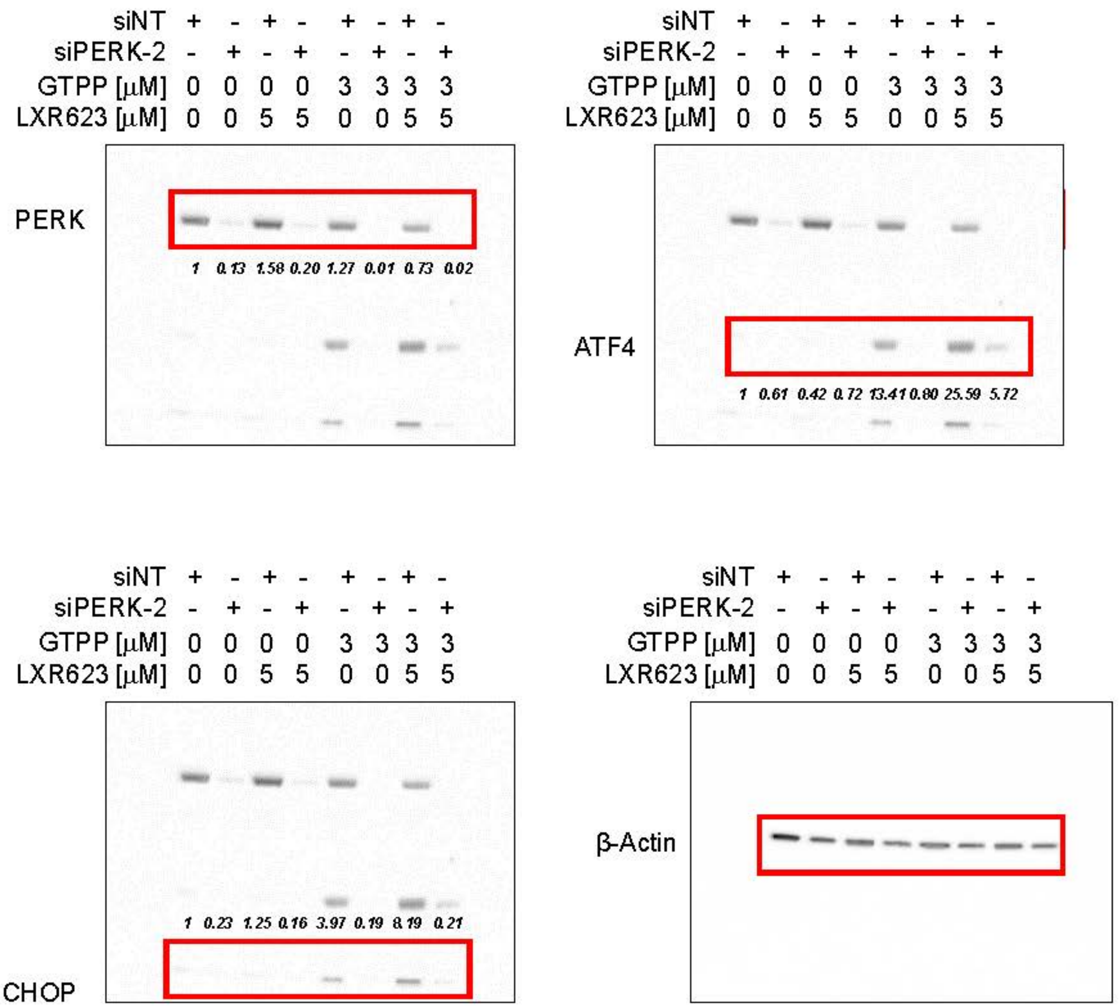
siNT + - + - + - + -
siPERK-2 - + - + - + - +
GTPP [μM] 0 0 0 0 3 3 3 3
LXR623 [μM] 0 0 5 5 0 0 5 5
PERK
1 0.13 1.58 0.20 1.27 0.01 0.73 0.02
siNT + - + - + - + -
siPERK-2 - + - + - + - +
GTPP [μM] 0 0 0 0 3 3 3 3
LXR623 [μM] 0 0 5 5 0 0 5 5
ATF4
1 0.61 0.42 0.72 13.41 0.80 25.59 5.72
siNT + - + - + - + -
siPERK-2 - + - + - + - +
GTPP [μM] 0 0 0 0 3 3 3 3
LXR623 [μM] 0 0 5 5 0 0 5 5
1 0.23 1.25 0.16 3.97 0.19 8.19 0.21
CHOP
siNT + - + - + - + -
siPERK-2 - + - + - + - +
GTPP [μM] 0 0 0 0 3 3 3 3
LXR623 [μM] 0 0 5 5 0 0 5 5
β-Actin

Figure 5h

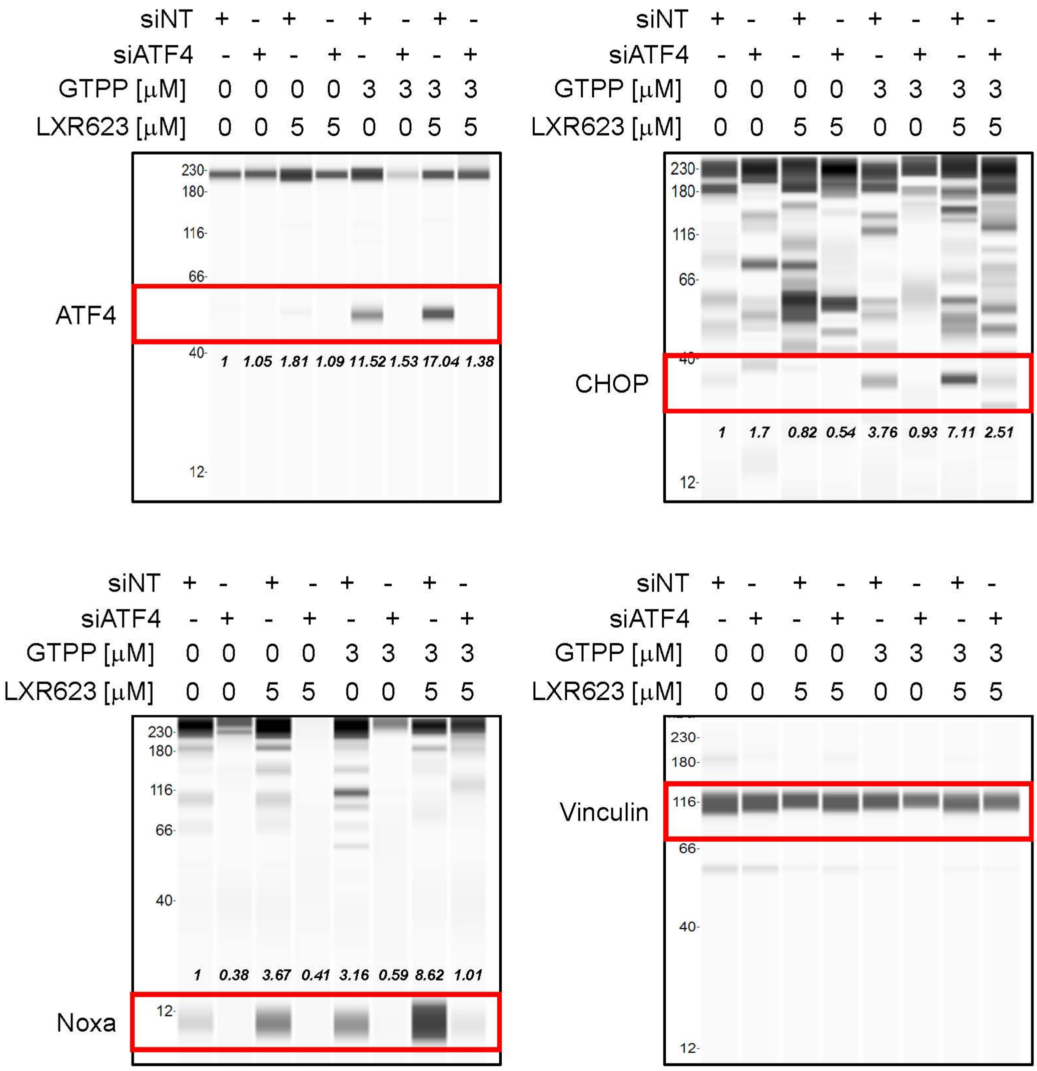
siNT + - + - + - + -
siATF4 - + - + - + - +
GTPP [μM] 0 0 0 0 3 3 3 3
LXR623 [μM] 0 0 5 5 0 0 5 5
ATF4
1 1.05 1.81 1.09 11.52 1.53 17.04 1.38
CHOP
1 1.7 0.82 0.54 3.76 0.93 7.11 2.51
Noxa
1 0.38 3.67 0.41 3.16 0.59 8.62 1.01
Vinculin
230
180
116
66
40
12

Supplementary Figure 1a

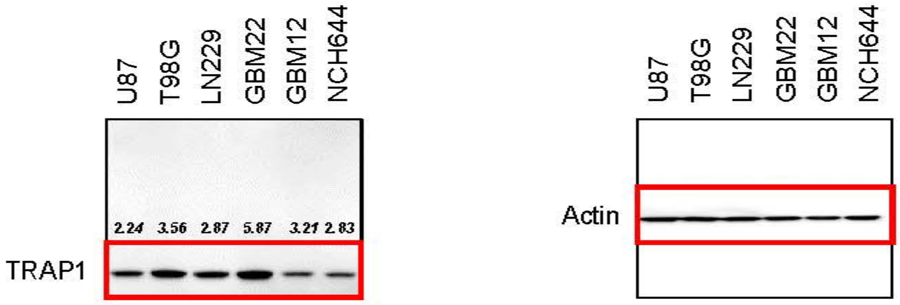

Supplementary Figure 1e

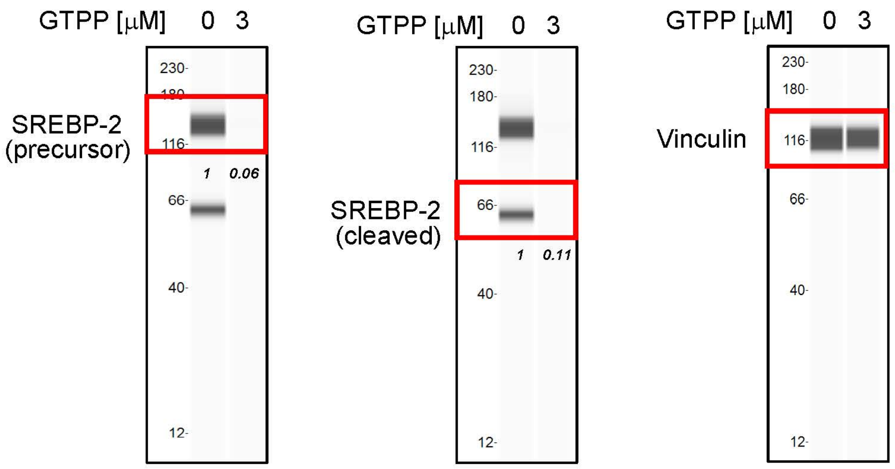

Supplementary Figure 1f

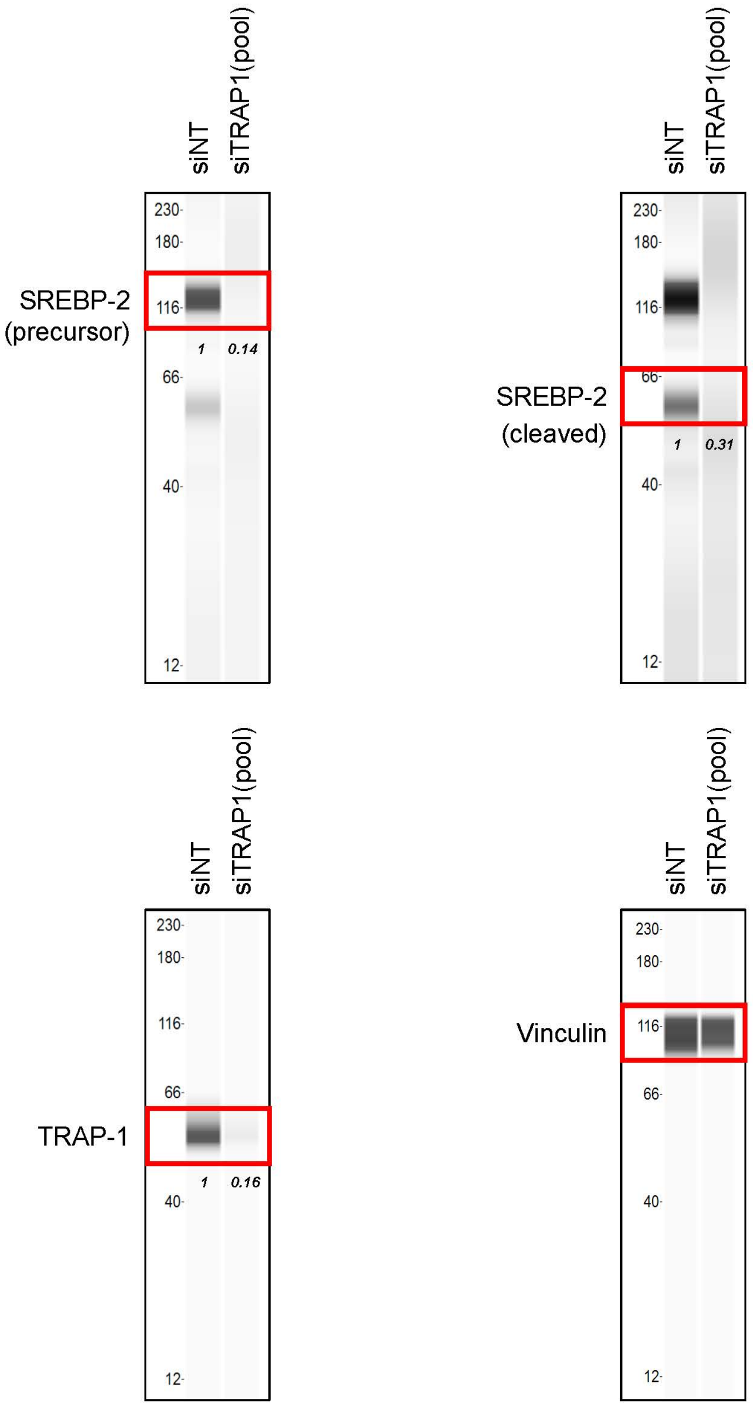

Supplementary Figure 1g

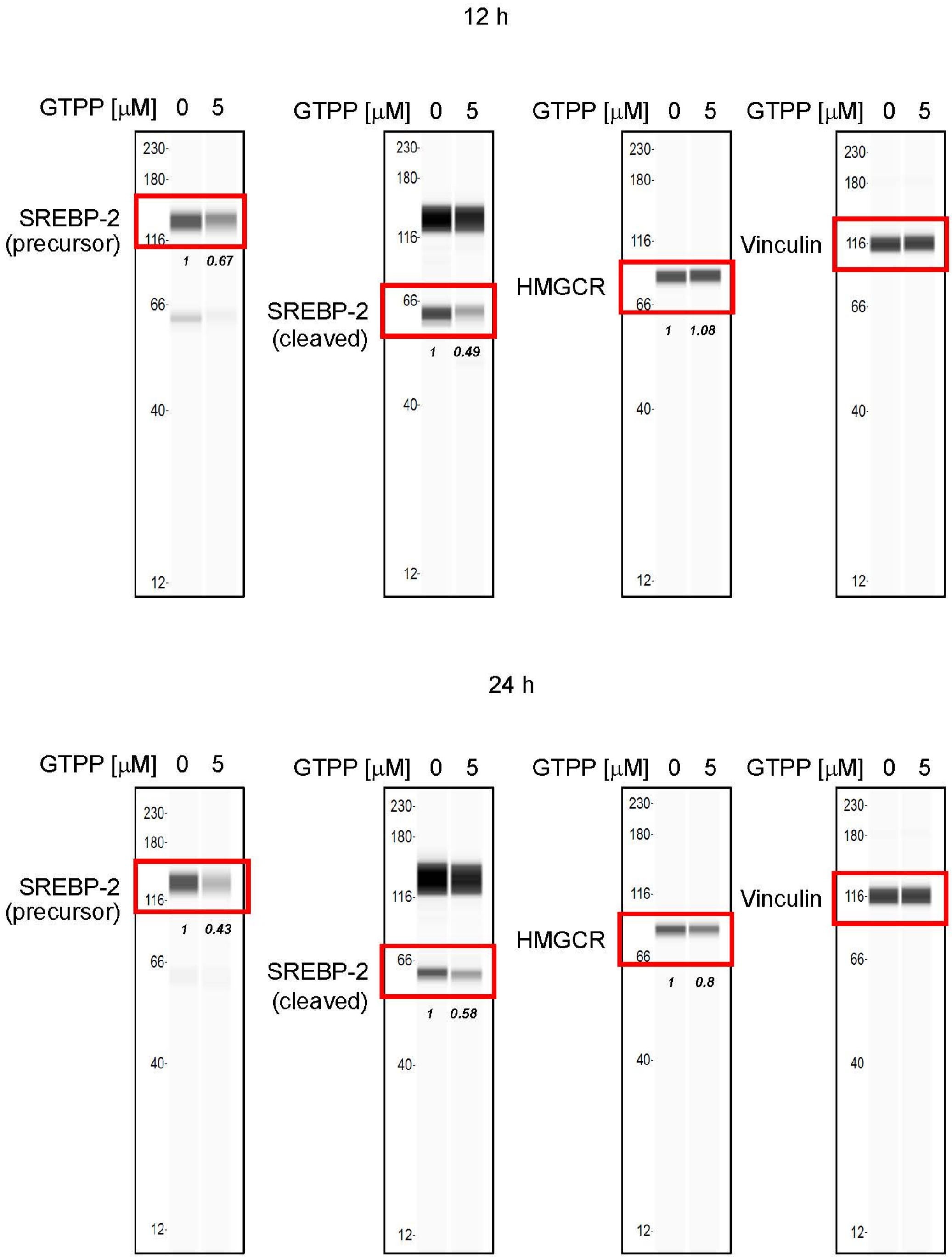

Supplementary Figure 1h

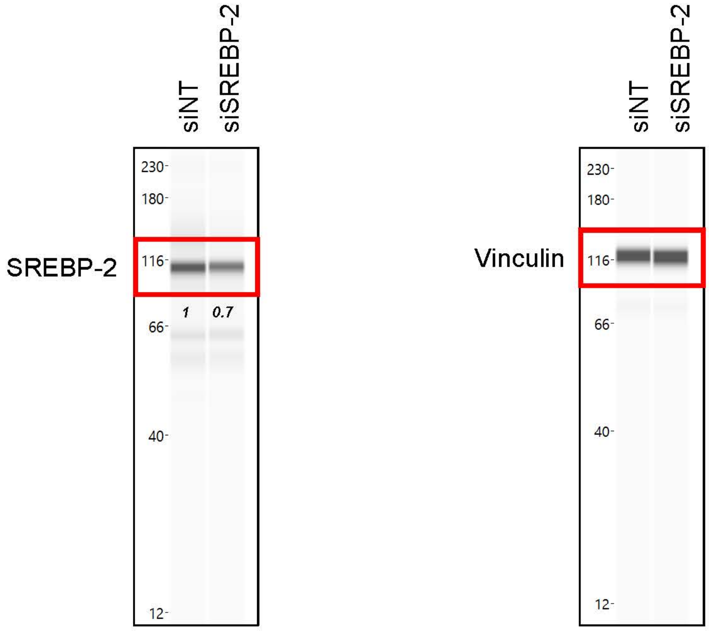

Supplementary Figure 3i

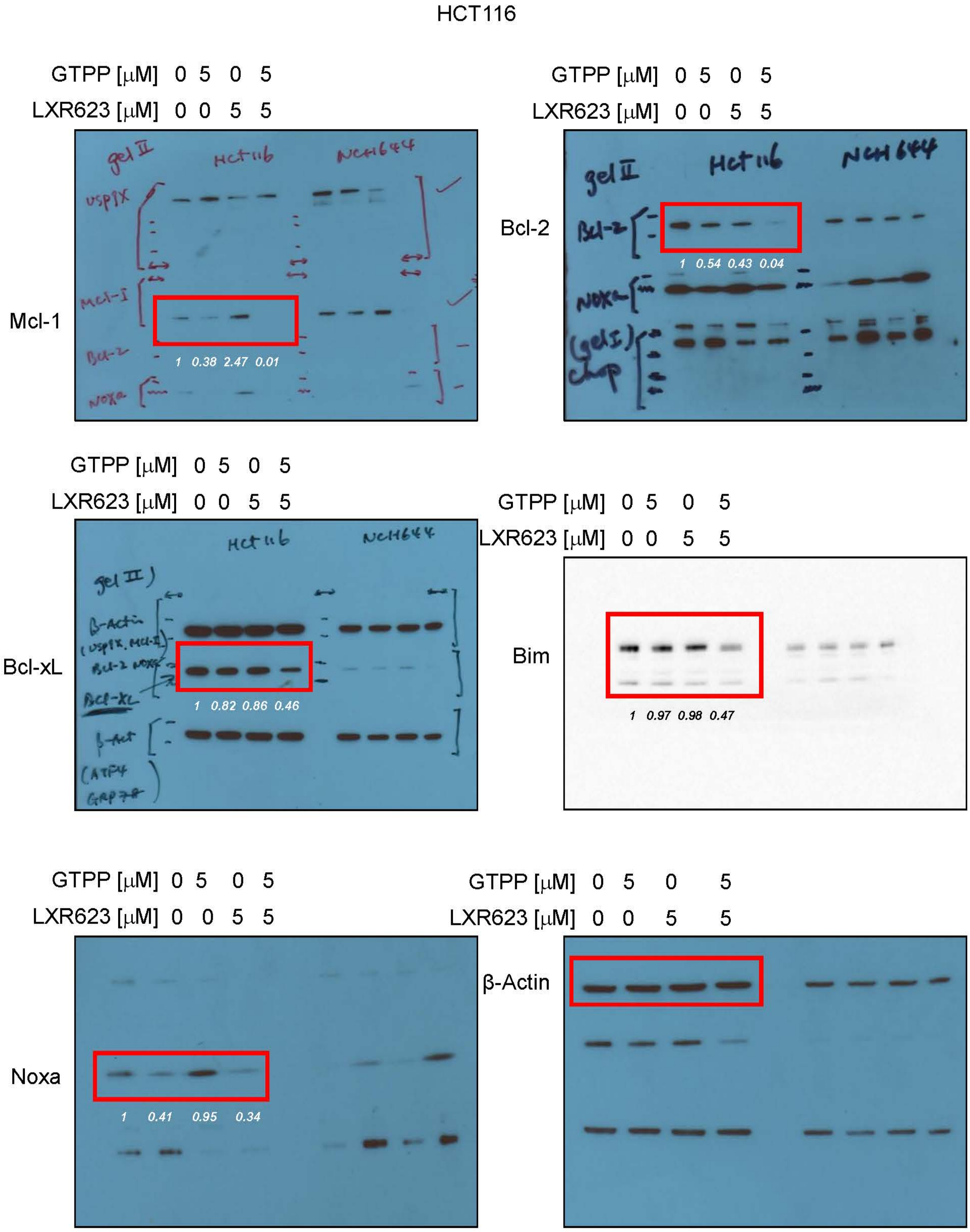

Supplement Figure 4a

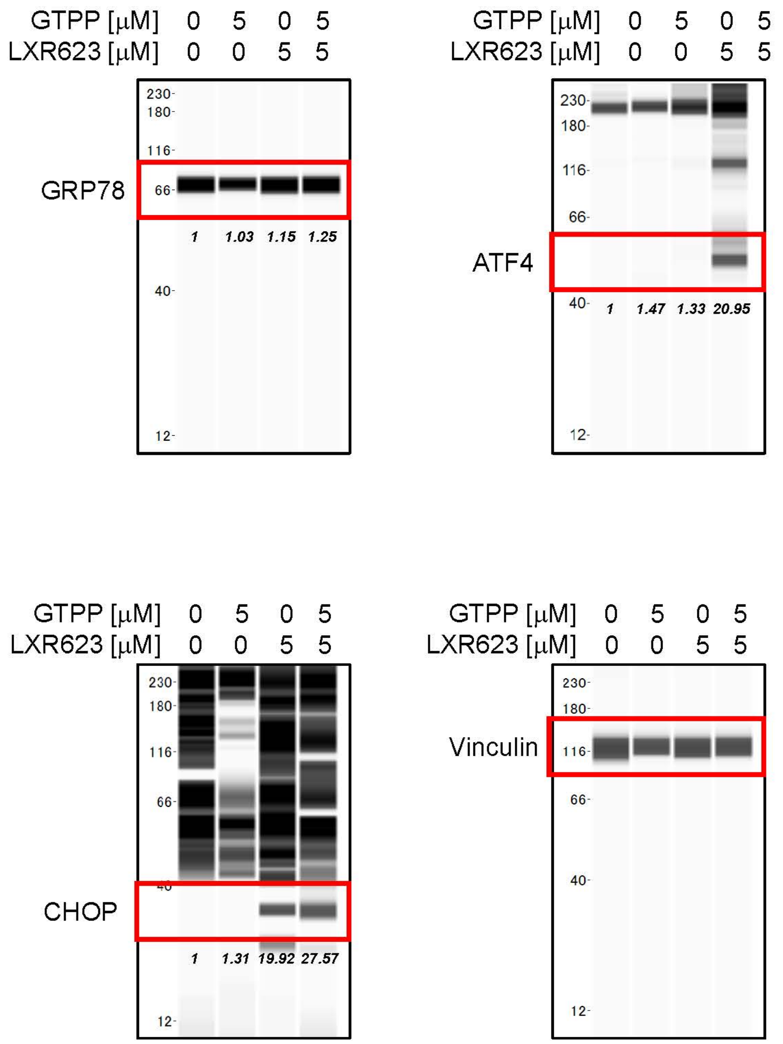

Supplement Figure 4b

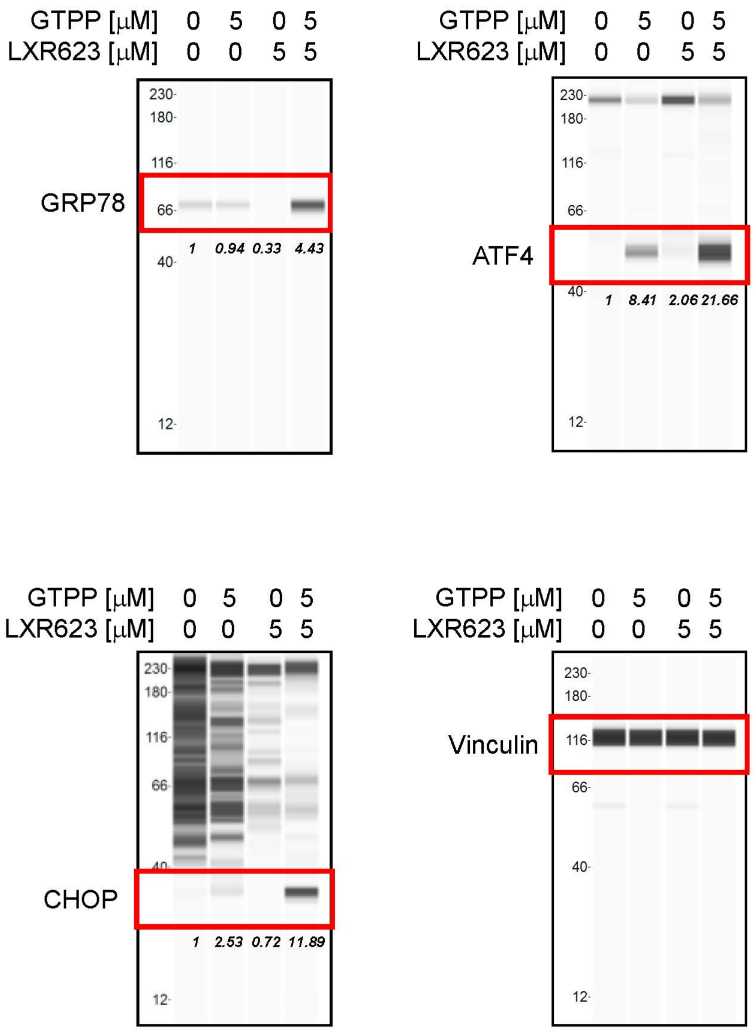

Supplement Figure 4c

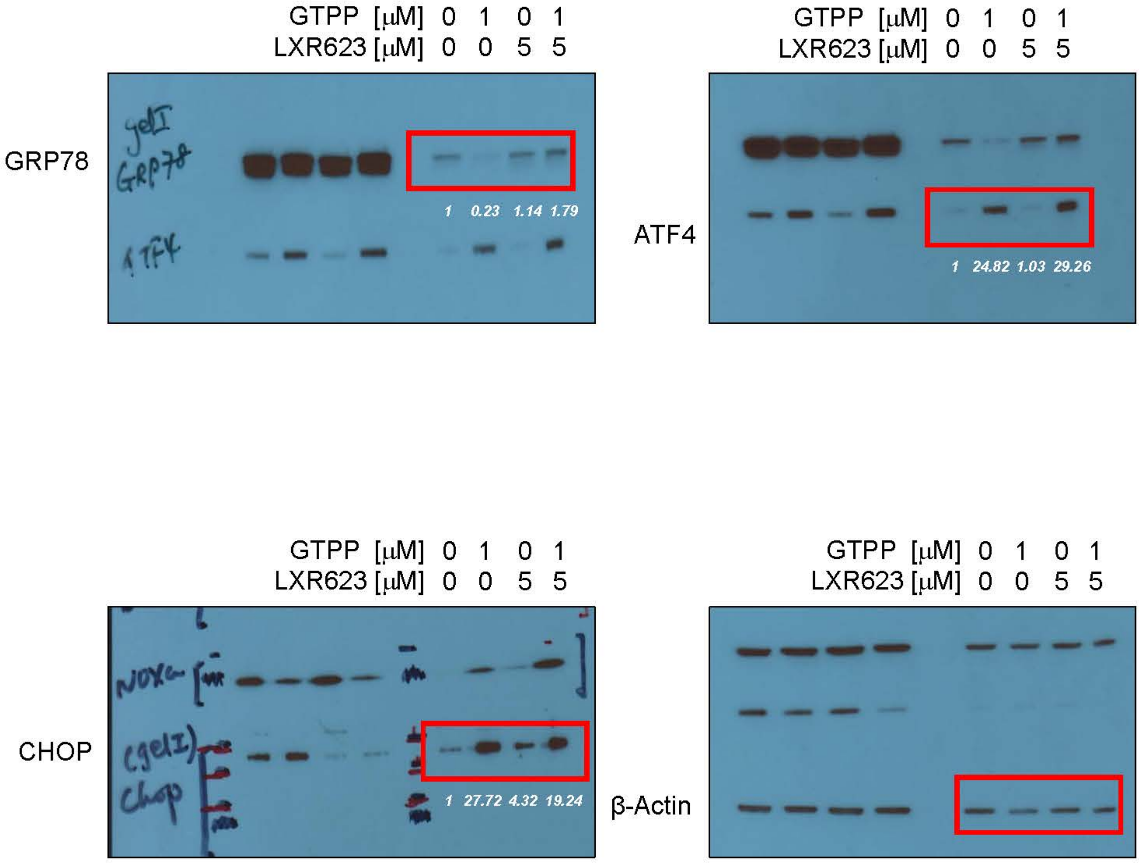

Supplement Figure 4d

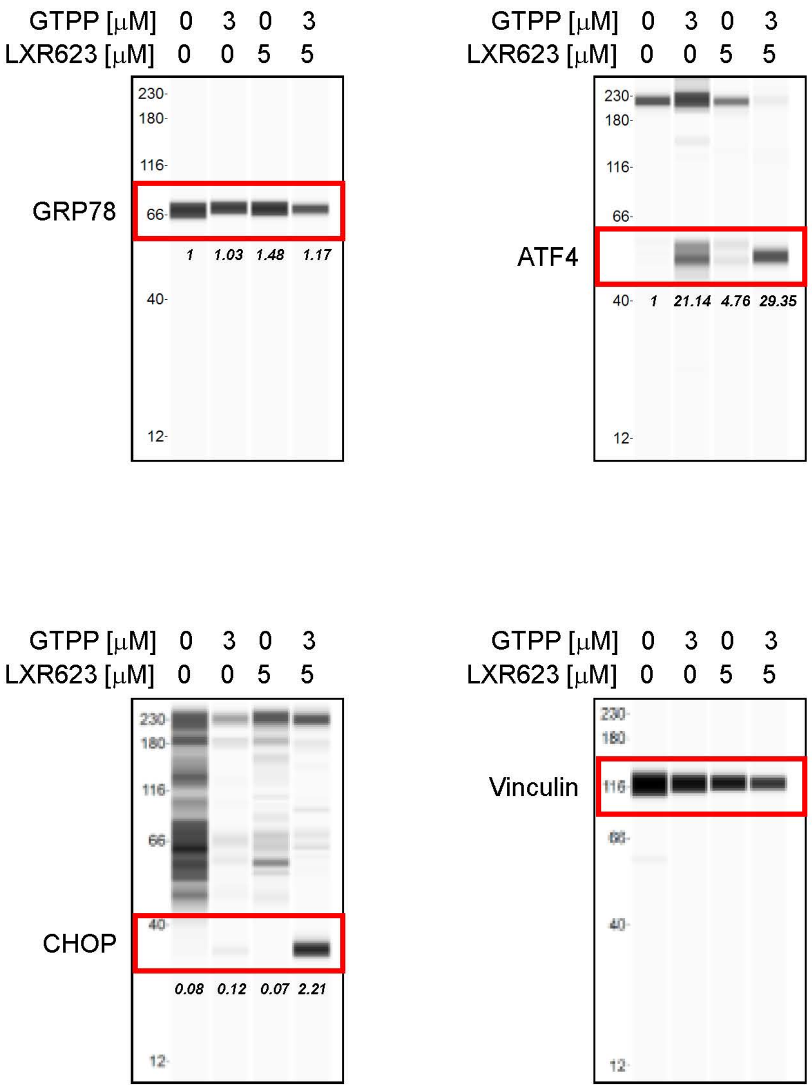

Supplement Figure 4e

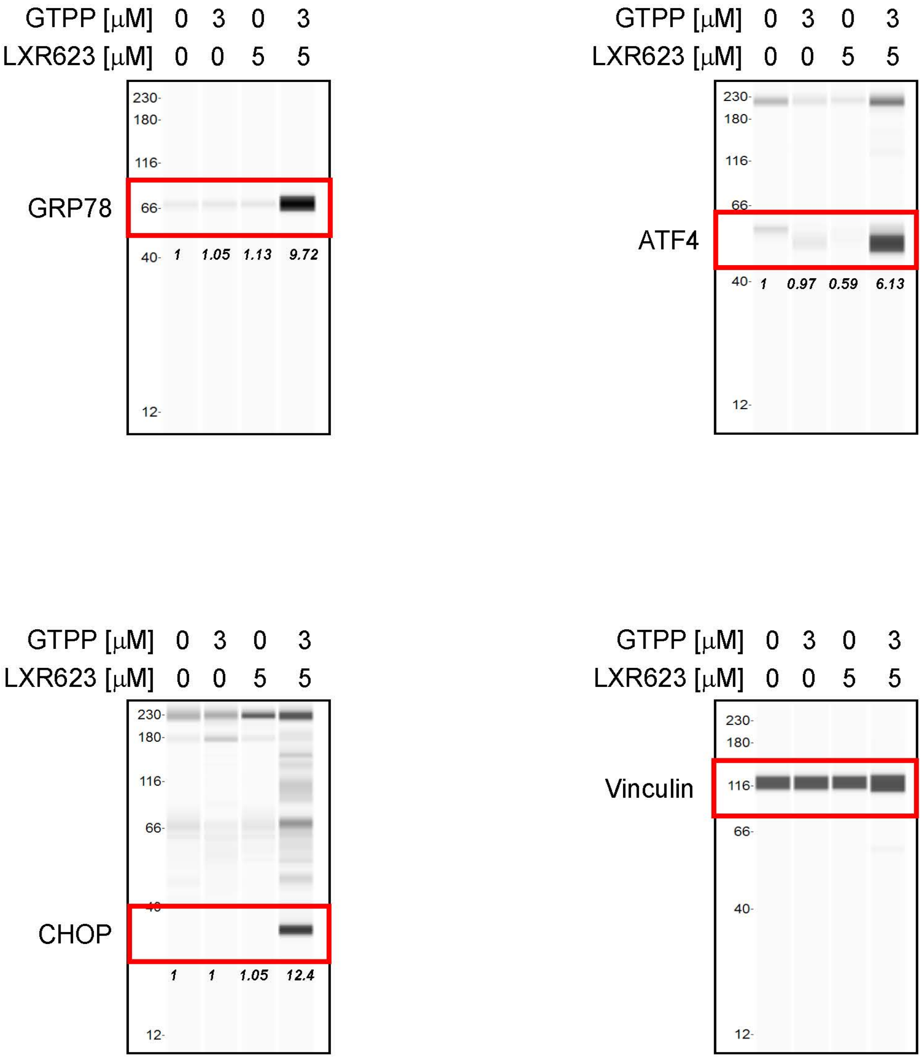

Supplement Figure 4f

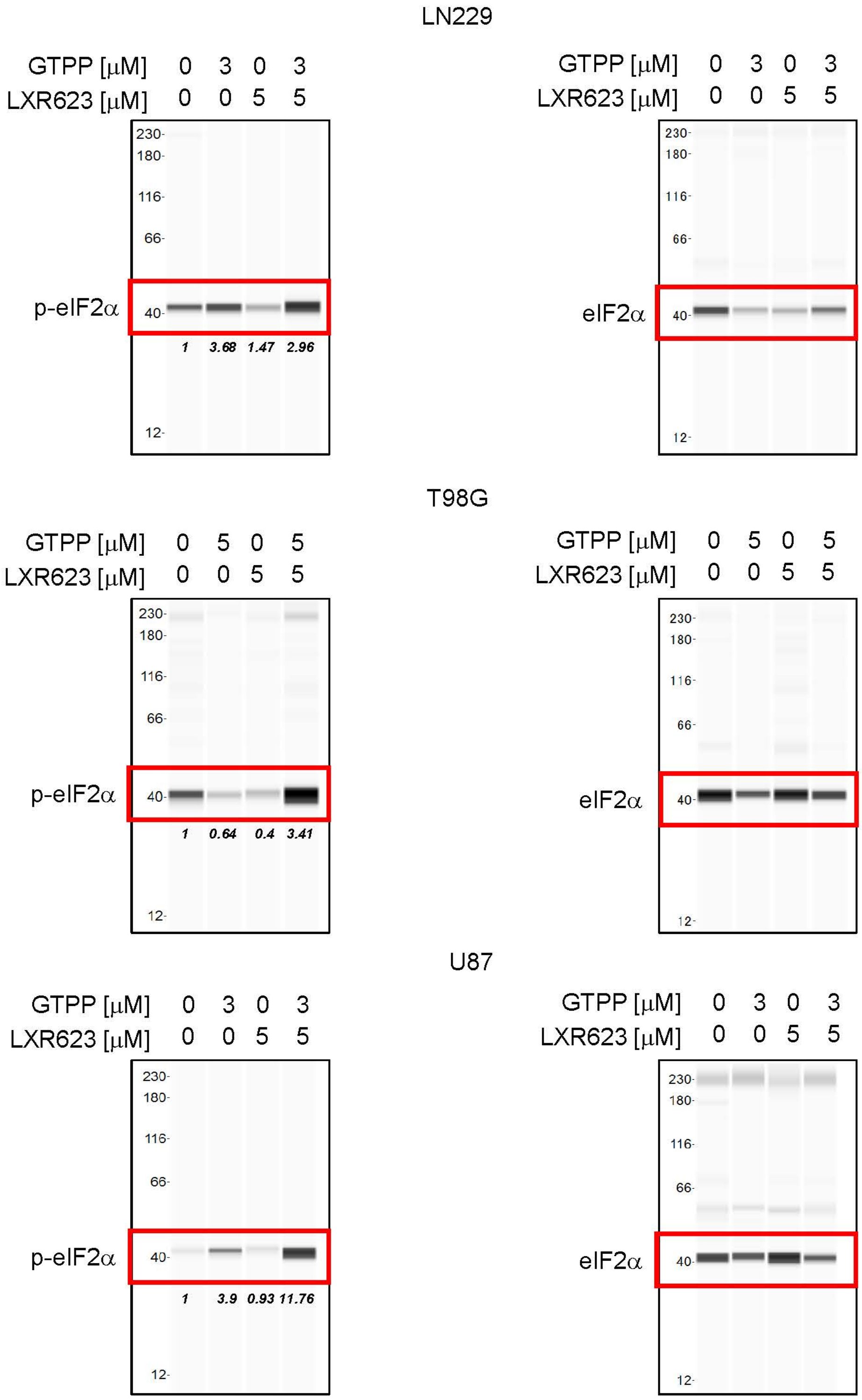

Supplement: Supplementary file 1 [file cancers-11-00788-s001.zip › cancers-512184-supplementary materials/cancers-512184-Supplementary Whole Blot.pdf]
